# Direct Magnetic Evidence, Functionalization, and Low-Temperature Magneto-Electron Transport in Liquid-Phase Exfoliated FePS_3_

**DOI:** 10.1021/acsnano.2c11654

**Published:** 2023-01-18

**Authors:** Lucía Martín-Pérez, Samara Medina Rivero, Manuel Vázquez Sulleiro, Alicia Naranjo, I. Jénnifer Gómez, María Luisa Ruíz-González, Andres Castellanos-Gomez, Mar Garcia-Hernandez, Emilio M. Pérez, Enrique Burzurí

**Affiliations:** † IMDEA Nanociencia C/Faraday 9, Ciudad Universitaria de Cantoblanco, 28049 Madrid, Spain; ‡ Department of Condensed Matter Physics, Faculty of Science, Masaryk University, Kotlářská 2, 61137 Brno, Czech Republic; § Departamento de Química Inorgánica, Universidad Complutense de Madrid, 28040 Madrid, Spain; ∥ 2D Foundry, Instituto de Ciencia de Materiales de Madrid (ICMM), Consejo Superior de Investigaciones Científicas (CSIC), 28049 Madrid, Spain; ⊥ Departamento de Física de la Materia Condensada and Condensed Matter Physics Center (IFIMAC), Universidad Autónoma de Madrid, 28049 Madrid, Spain

**Keywords:** liquid-phase exfoliation, magnetic van der Waals, two-dimensional, electron transport, FePS_3_, magnon

## Abstract

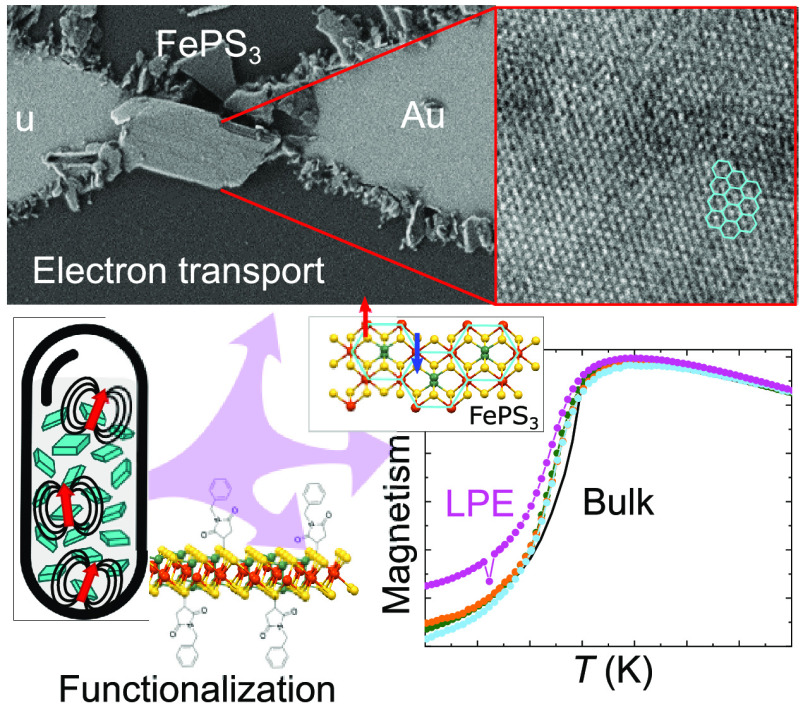

Magnetism and the existence of magnetic order in a material is determined by its dimensionality. In this regard, the recent emergence of magnetic layered van der Waals (vdW) materials provides a wide playground to explore the exotic magnetism arising in the two-dimensional (2D) limit. The magnetism of 2D flakes, especially antiferromagnetic ones, however, cannot be easily probed by conventional magnetometry techniques, being often replaced by indirect methods like Raman spectroscopy. Here, we make use of an alternative approach to provide direct magnetic evidence of few-layer vdW materials, including antiferromagnets. We take advantage of a surfactant-free, liquid-phase exfoliation (LPE) method to obtain thousands of few-layer FePS_3_ flakes that can be quenched in a solvent and measured in a conventional SQUID magnetometer. We show a direct magnetic evidence of the antiferromagnetic transition in FePS_3_ few-layer flakes, concomitant with a clear reduction of the Néel temperature with the flake thickness, in contrast with previous Raman reports. The quality of the LPE FePS_3_ flakes allows the study of electron transport down to cryogenic temperatures. The significant through-flake conductance is sensitive to the antiferromagnetic order transition. Besides, an additional rich spectra of electron transport excitations, including secondary magnetic transitions and potentially magnon-phonon hybrid states, appear at low temperatures. Finally, we show that the LPE is additionally a good starting point for the mass covalent functionalization of 2D magnetic materials with functional molecules. This technique is extensible to any vdW magnetic family.

Magnetism is intrinsically intertwined with dimensionality. The existence itself of a magnetic order transition is determined by the number of dimensions defining a material. Two-dimensional (2D) materials can only undergo a true long-range magnetic phase transition for an Ising configuration of spins. The exact model was solved by Onsager.^1^ On the other hand, 2D XY systems can only show quasi-long-range order, theoretically modeled by Kosterlitz and Thouless,^2^ with the appearance of magnetic vortices.

Besides magnetic order, some exotic magnetic phenomena may appear at low dimensions, like topological magnetic skyrmions^3,4^ and quantum phases, like quantum spin liquids, induced by strong quantum fluctuations in the 2D limit.^5^ Moreover, the combination of conductivity and magnetism in a single 2D material may lead to phenomena like electrostatic gating of the magnetism to provide atomic-flat spintronics devices.^4,6,7^

The recent emergence of magnetic layered van der Waals (vdW) materials greatly expanded the number and variety of available 2D magnetic candidates.^8^ van der Waals materials are stacks of 2D covalent lattices weakly held together by vdW interactions. These cleavable materials can be easily exfoliated to obtain truly atomically thin layers. Nowadays, several reports unambiguously confirm the persistence of a magnetically ordered phase down to the monolayer in the ferromagnetic CrGeTe_3_,^9^ Fe_3_GeTe_2_,^6^ VSe_2_,^10^ CrI_3_,^11,12^ and the antiferromagnetic FePS_3_,^13,14^ among other examples. The magnetism of individual exfoliated flakes cannot be, however, easily studied by means of conventional magnetometry techniques. Only a few state-of-the-art nano-SQUID magnetometers promise enough magnetic sensitivity to explore the atomic layer.^15,16^ Instead, the presence of a magnetic order has been studied so far by alternative methods like magneto-optic Kerr effect (MOKE)^9,12^ and Raman spectroscopy.^13,14^ However, MOKE is not applicable for antiferromagnetic materials, whereas Raman provides only indirect information on structural changes that may arise upon the time-reversal symmetry breaking in a magnetic transition. Therefore, albeit being an interesting tool, Raman does not provide a direct magnetic evidence and offers a limited view that does not clearly separate magnetism from lattice structure.

A paradigmatic example of these limitations is the FePS_3_ vdW trichalcogenide. The FePS_3_ magnetic structure involves a 2D honeycomb lattice of Fe^2+^ ions magnetically connected by superexchange via two equivalent S atoms, as seen in Figure 1c.^13,17−19^ Bulk FePS_3_ becomes an Ising antiferromagnet at around 118 K with the spins pointing out of plane.^20,21^ Lately, FePS_3_ has aroused great interest since the discovery of emergent superconductivity induced by pressure-driven spin crossover.^22^ Moreover, its cleavage energy is lower than graphite,^23^ which facilitates the exfoliation of FePS_3_ down to thin flakes and the study of its magnetism.^13,14^ The appearance/disappearance and splitting of specific Raman modes with temperature in the vicinity of the well-known bulk Néel transition was used as an indirect fingerprint of a magnetic transition in FePS_3_ thin flakes. The tracking of those peaks proved to be very sensitive however to the laser excitation wavelength, the local environment and, more importantly, the laser power, which is known to induce local heat in samples thus potentially disturbing the magnetic order. Moreover, it may also be sensitive to alterations in the lattice structure around the Fe as a result of the exfoliation, like strain or damage, which may affect the vibrational spectra but are not straightforwardly connected to the magnetic transition. As an example, a very recent report shows that the Néel temperature (*T*_N_) in FePS_3_ can drastically decrease with slight strain^24^ and is not necessarily related to the number of layers.

**Figure 1:**
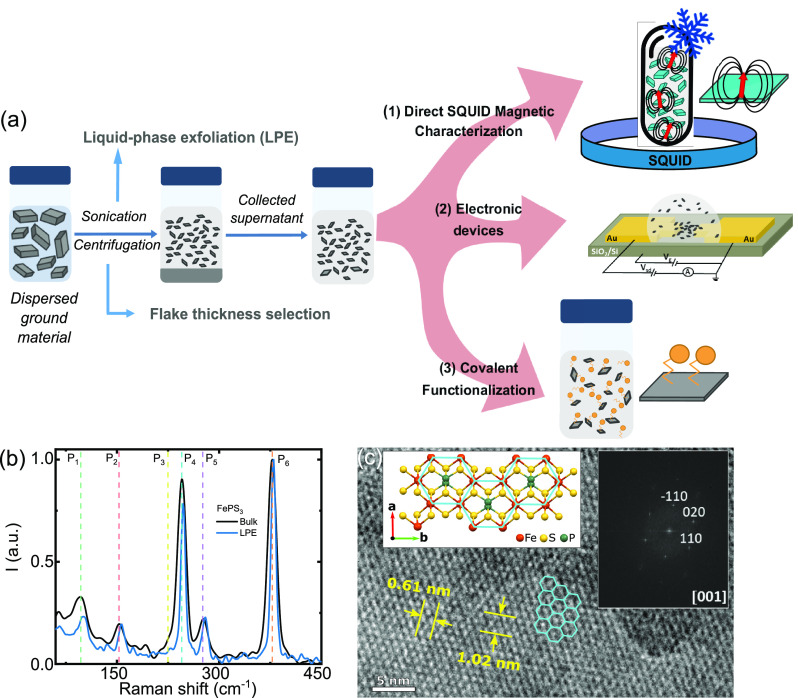
(a) Schematic of the two-step LPE process. Bulk material is ground in an agate mortar and thereafter dispersed in *i*PrOH at a 1 mg·mL^–1^ concentration. The dispersion is sonicated in an ultrasonic bath and centrifuged following a liquid-cascade-centrifugation methodology with increasing centrifugation speed (ω_1–4_ = 1000, 3000, 5000, and 7000 rpm, respectively). LPE exfoliation allows (1) a direct SQUID magnetic characterization in solvent, (2) the fabrication of electronic devices, and (3) the mass covalent functionalization of FePS_3_. (b) Normalized Raman spectra (λ_exc_ = 785 nm) of bulk (gray line) and LPE (blue line) FePS_3_. (c) HRTEM image of a LPE FePS_3_ flake with the corresponding SAED pattern. The inset shows the crystal packing structure of FePS_3_ along *c* axis. The Fe atoms form a honeycomb lattice.

The combination of all these factors resulted in a variety of different Raman spectra in the literature and more importantly showed a disagreement in key aspects of the magnetic properties of exfoliated FePS_3_. While Wang et al. show a drop in the *T*_N_ with decreasing thickness of the sample,^14^ Lee at al. show no significant variation of *T*_N_ down to the monolayer limit.^13^

Here, we present an alternative approach to directly study the magnetism of thin flakes of magnetic vdW materials, including antiferromagnets. The quality of the flakes additionally allows to study the electronic properties and perform large-scale covalent functionalization, as summarized in Figure 1a. We perform liquid-phase exfoliation (LPE) of FePS_3_ in *i*PrOH.^25,26^ We show that the exfoliation proceeds smoothly without the help of surfactant species or intercalating agents used in other liquid exfoliation methods.^27−29^ Thousands of flakes, with a narrow and tunable thickness distribution, remain dispersed in *i*PrOH which can be easily quenched and measured in conventional SQUID magnetometers in solvent, avoiding reaggregation into solid-state.^30^ We obtain a direct evidence of the antiferromagnetic transition in thin flakes (5–9 layers) of FePS_3_. The Néel temperature steadily decreases with decreasing thickness in the exfoliated flakes in contrast with some reports in literature based on Raman measurements.^13^

In addition, we take advantage of the suspended flakes to fabricate FePS_3_ nanoscale transistors by dielectrophoresis.^25,26^ We observe a sizable conductance in the few-layers FePS_3_ flakes. Moreover, we show that the antiferromagnetic transition and a second low-temperature magnetic transition are translated into perturbations in the electrical current and the appearance of low-energy current excitations. Some of these excitations could be identified with the presence of magnon quasiparticles.

Finally, we show the covalent functionalization of the thin flakes in solution with small organic molecules. Functionalization is possible in the clean FePS_3_ surfaces thanks to the absence of chemical exfoliating agents. We show that the covalent functionalization does not significantly alter the magnetic properties of the FePS_3_ layers.

## Results and Discussion

### LPE Exfoliation and Structural Analysis

Figure 1a shows a schematic of the LPE process used to obtain FePS_3_ few-layers flakes.^25,26,31^ The bulk material is initially ground in an agate mortar to obtain a powder that is dispersed in *i*PrOH at a concentration of 1 mg·mL^–1^. The resultant dispersion is then exfoliated following a two-step process: (i) 40 min sonication in an ultrasonic bath kept at 20 °C, followed by (ii) a centrifugation cascade (30 min, 20 °C, Beckman Coulter Allegra X-15R, FX6100 rotor, radius 9.8 cm) with increasing centrifugal speed, (ω_1–4_ = 1000 (110), 3000 (986), 5000 (2739) and 7000 (5369) rpm (g)). A fraction of the resultant gray-colored supernatant is kept after each centrifugation. The remainder supernatant is centrifuged at a higher speed to obtain the consecutive samples (ω_1_, ω_2_, ω_3_, ω_4_). Increasing centrifugation speed is expected to provide thinner flakes on average.^32,33^ All sediments are discarded.

LPE FePS_3_ thin flakes are characterized by Raman spectroscopy to explore the integrity of the material after the exfoliation process. Figure 1b shows the Raman spectra of bulk and ω_1_ (1000 rpm) LPE flakes measured at room temperature with a 785 nm laser excitation wavelength. LPE samples are prepared by filtering the supernatant collected after the ω_1_ centrifugation step (see Methods). The final spectrum is averaged over 144 measurements obtained in a 14 μm × 14 μm area covered with FePS_3_ flakes to average out morphological diversities as a result of the LPE process; see optical images of the area in Figure S2 of the Supporting Information (see Methods for details). The characteristic peaks of bulk FePS_3_ appear below 600 cm^–1^.^13,23^ The most intense Raman band at 378 cm^–1^ (P_6_) is assigned to the P–S symmetric stretching vibration. The 278 cm^–1^ (P_5_), 245 cm^–1^ (P_4_), and 219 cm^–1^ (P_3_) peaks correspond to PS_3_ deformations,^28,34,35^ while the peak at 155 cm^–1^ (P_2_) is assigned to the P–P single bond stretching.^36^ The low-frequency broad band around 93 cm^–1^ (P_1_) involves vibrations of the transition-metal atom.^13,14,17,34,35,37^ The Raman spectra of the LPE flakes present the same characteristic modes at roughly the same energies. Only a slight blueshift (∼3 cm^–1^) is observed for P_1_; see Figure S1 in the Supporting Information for the Raman fittings. A similar result is observed for higher centrifugation speeds, as seen in Figures S3 and S4 in the Supporting Information. No significant shifts are expected for metal phosphorus trichalcogenides due to the weaker interlayer vdW interactions when compared to other 2D materials.^38^ Larger centrifugation speeds lead to a slight P_4_ redshift and P_6_ blueshift associated with the thinning down of the sample^13^ (see Figure S5 in the Supporting Information). The lattice structure of thin flakes is therefore roughly preserved after LPE and in ambient conditions.

Additional proof of the lattice integrity after LPE is provided by high resolution transmission electron microscopy (HRTEM). Figure 1c shows a HRTEM image of a representative FePS_3_ LPE nanoflake (see Methods for additional details and Figure S6 for additional images). The flake exhibits a lamellar morphology in which the Fe honeycomb lattice can be clearly distinguished. No significant distortions nor defects can be observed in the periodicity of the lattice. The measured lattice spacings are 0.61 and 1.02 nm (see Figure 1c), in agreement with the monoclinic FePS_3_ cell along the [001] zone axis.^39^ The corresponding FTT, shown in the inset of Figure 1c, confirms the above orientation. The results demonstrate the high crystallinity of the exfoliated nanosheets after LPE.

**Figure 2:**
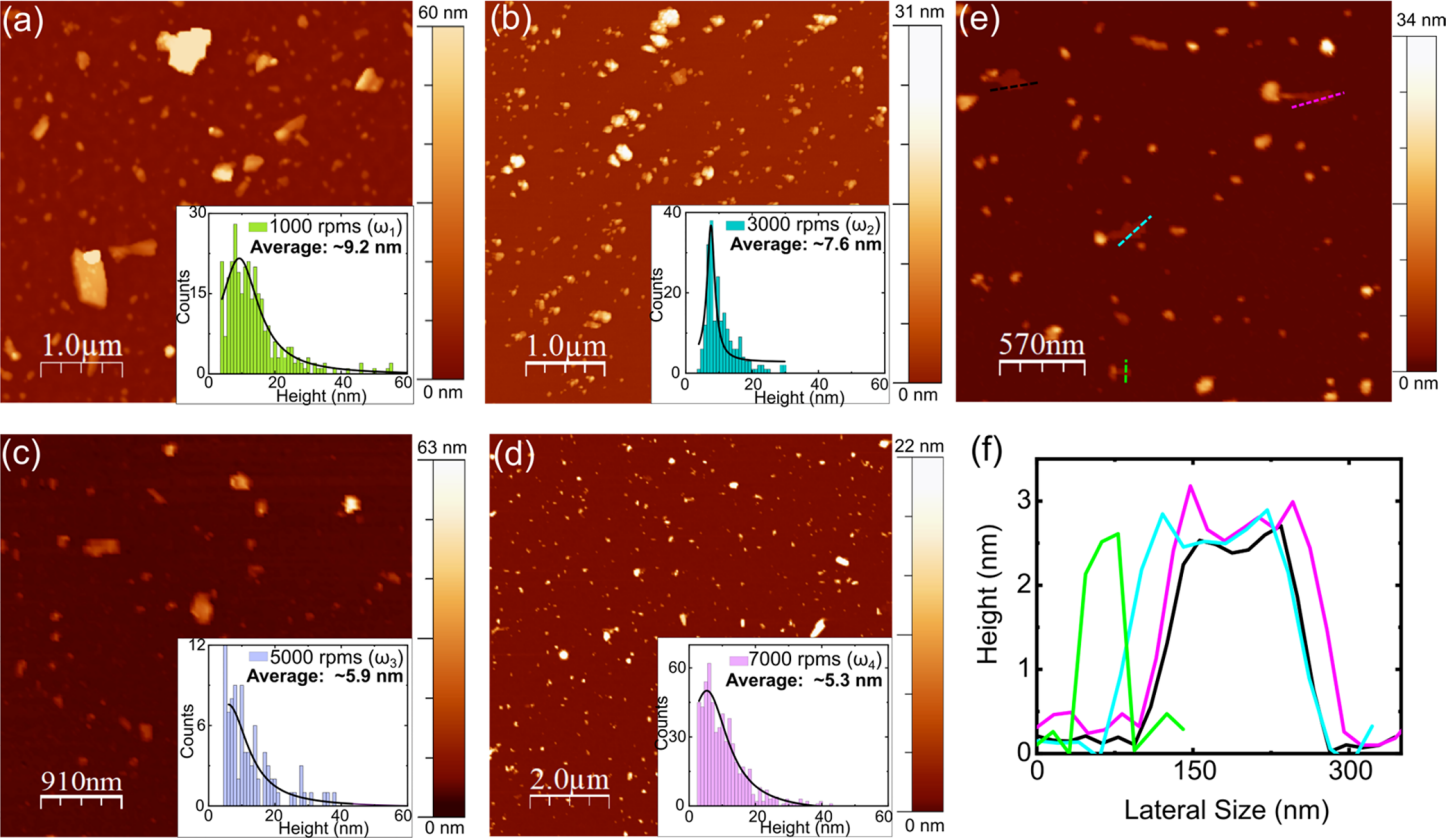
AFM topographic characterization of LPE FePS_3_ nanoflakes obtained at different centrifugation speeds: (a) ω_1_ = 1000, (b) ω_2_ = 3000, (c) ω_3_ = 5000, and (d) ω_4_ = 7000 rpm. The insets show the flake thickness distribution. The fitting to a Lorentzian curve provides flake modal thicknesses of 9.2 nm (ω_1_), 7.6 nm (ω_2_), 5.9 nm (ω_3_), and 5.3 nm (ω_4_), respectively. (e) Closer view of nanoflakes obtained in sample ω_4_. (f) Height profile of four selected FePS_3_ flakes in (e).

The morphology and size distribution of the LPE FePS_3_ nanoflakes are studied by atomic force microscopy (AFM). Samples are prepared by spin-coating of the corresponding dispersion on mica foil and dried in air (see Methods). Figure 2a–d shows the AFM images of the FePS_3_ nanoflakes obtained after the four consecutive centrifugation steps. Several micro and nanoscale flakes can be observed. Figure 2e,f shows some representative nanoflakes (ω_4_) with their corresponding thickness profile (∼2.5 nm). The statistical analysis of the images provides a flake modal thickness of 9.2 nm (ω_1_), 7.6 nm (ω_2_), 5.9 nm (ω_3_), and 5.3 nm (ω_4_), respectively. Therefore, the higher the centrifugation speed, the lower the average thickness of the resultant nanosheets; see Figure S4 in the Supporting Information for additional AFM data and statistical analysis. These results quantitatively agree with reports in literature for other wet methods.^28^ Note that the significant advantage of the present method is that no electrolytes, intercalating agents, additional washing steps, nor temperature annealing^27−29,40,41^ of the collected supernatant have been employed, leaving the material unaltered and the surface clean for further chemical functionalization.

The equivalent number of layers can be roughly estimated by considering a 0.64 nm interlayer spacing^14,23^ and a typical 1.2–1.3 nm correction to account for adsorbed solvent molecules, impurities, or moisture trapped between layers.^42,43^ The resulting average number of layers is 6–7 (ω_1_), 5–6 (ω_2_), and 4–5 (ω_3_, ω_4_), respectively.^28^ Note that these values may be slightly overestimated since the flake identification routine picks the highest point in the flake (see Figure S7 in the Supporting Information).

The lateral dimensions of the flakes have been analyzed as well from the AFM images (see Figures S8 and S9 in the Supporting Information). The statistical analysis shows that the modal area and perimeter of the flakes slightly decrease with centrifugation speed, as expected by the mass selectivity of the centrifugation cascade. The perimeter of the flakes remains in the few hundreds nanometer scale.

### Direct Magnetic Characterization

**Figure 3:**
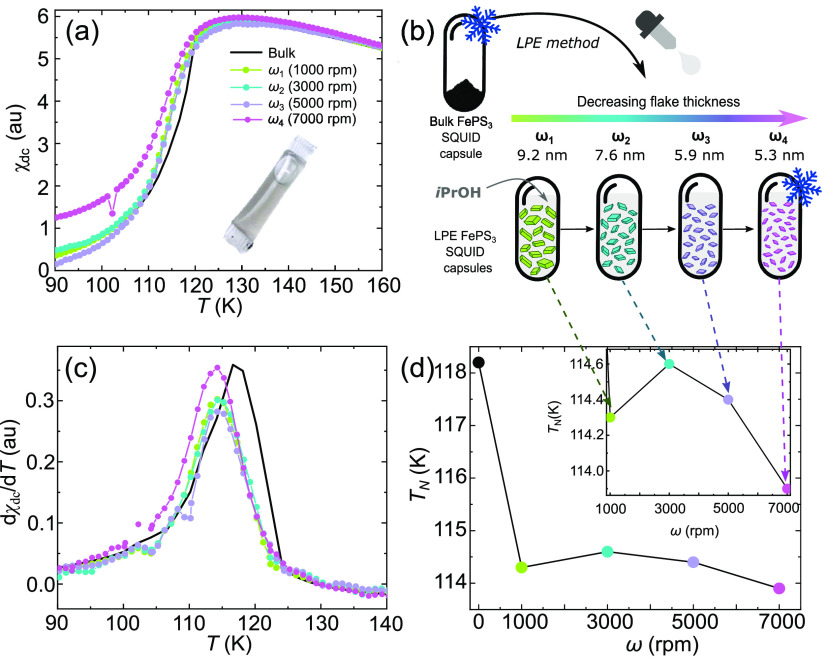
(a) Magnetic susceptibility measured as a function of temperature in a bulk reference sample and exfoliated samples ω_1_, ω_2_, ω_3_, and ω_4_ corresponding to average flake thicknesses of 9.2, 7.6, 5.9, and 5.3 nm, respectively. The antiferromagnetic transition is preserved in the thin exfoliated flakes. The inset shows an optical image of a representative SQUID capsule with LPE FePS_3_. (b) Graphical representation of the SQUID magnetic measurements. LPE FePS_3_ flakes with different average thickness are quenched in *i*PrOH in the SQUID mangetometer. (c) First derivative of χ with respect to the temperature (dχ/d*T*). The Néel temperature (*T*_N_) is shifted from 118 K in powder down to approximately 114 K in the exfoliated flakes. (d) *T*_N_ as a function of the centrifugation speed. A sharp drop in *T*_N_ is observed from bulk to ω_1_ (average flake thickness: 9.2 nm), while a smaller yet clear drop in *T*_N_ is observed down to ω_4_, that is, the samples with the lowest average thickness (∼5.3 nm). See inset for a magnification of the data points corresponding to the exfoliated samples.

The magnetic properties of the FePS_3_ few-layer flakes can be studied, taking advantage of LPE, by direct SQUID magnetic measurements of the flakes suspended in the solvent (see schematics in Figure 3b). Figure 3a shows the magnetic susceptibility (χ) measured as a function of temperature in bulk material (nonexfoliated powder) as well as the four thickness-dependent LPE samples ω_1_, ω_2_, ω_3_, and ω_4_. The measurements are carried out in a SQUID magnetometer from Quantum Design (see Methods). For exfoliated samples (ω_1_, ω_2_, ω_3_, and ω_4_), a *i*PrOH microdroplet (∼330 μL) containing dispersed, freshly exfoliated flakes is enclosed in a plastic diamagnetic capsule, as seen in Figure 3. The material in the capsule is briefly resonicated and immediately quenched at cryogenic temperatures within the SQUID magnetometer to avoid reaggregation. All SQUID measurements are carried out at temperatures below 170 K to ensure that *i*PrOH remains frozen (melting point 184 K) during the measuring process, and therefore, there is no relative movement of flakes that could perturb the magnetic measurement. The paramagnetic and diamagnetic contributions originated from the frozen *i*PrOH and the plastic capsule are subtracted by measuring a control capsule containing only *i*PrOH; see Figure S10 in the Supporting Information for the raw magnetic measurements and the correction of background contributions to the susceptibility.

First, the bulk FePS_3_ susceptibility is measured as a reference (solid line in Figure 3a). A sharp drop in χ is indicative of a paramagnetic to antiferromagnetic state transition with a Néel temperature of *T*_N_ = 118 K, in agreement with previous reports in the literature.^20,21^ Interestingly, exfoliated samples ω_1_, ω_2_, ω_3_, and ω_4_ present a similar, slightly shifted para- to antiferromagnetic phase transition at comparable temperatures (see Figure 3a). This result represents a direct magnetic evidence showing the antiferromagnetic transition in exfoliated FePS_3_ few-layers flakes (<9 nm). It is interesting to note that the width of the magnetic transition is not significantly broader than in bulk. This may be indicative of a comparable distribution of flake thickness between bulk and LPE flakes. Only a small broadening of the low temperature tail is observed for sample ω_4_, probably pointing to the presence of a significant population of flakes thinner than the average, as hinted by AFM in Figure 2.

The Néel temperature in the exfoliated samples is obtained as the maximum in the first derivative of the susceptibility with temperature (dχ/d*T*), as shown in Figure 3c. A clear drop in *T*_N_ down to 114.3 K is observed for sample ω_1_. Therefore, the Néel temperature in flakes with average 9–5 layers is roughly 4 K lower than nonexfoliated powder samples. This direct magnetic evidence therefore confirms the scenario suggested by previous Raman methods on mechanically exfoliated flakes.^14^ The dependence between *T*_N_ and the number of layers seems in quantitative agreement although the drop seems softer than predicted by Raman. In addition, a less pronounced though clear drop of *T*_N_ with increasing ω can be observed for LPE samples ω_1_, ω_2_, ω_3_, and ω_4_, as seen in Figure 3d and inset. This trend can be understood when inspecting the AFM statistical analysis performed in the four samples (see Figure 2 and Figure S7 in the Supporting Information). Only a minor variation in the thickness distribution (<4 nm, 3–4 layers) can be observed between samples, in agreement with previous reports where thin flakes were obtained by chemical exfoliation.^28^ Therefore, only minor variations in *T*_N_ could be expected. Note also that ω_1_ seems to slightly deviate from the general trend. This behavior is reproducible in different sets of samples and it so far not well understood.

The susceptibility measurements indicate, therefore, that while magnetic order is essentially dominated by intralayer exchange interactions, the interlayer interactions may play a minor though non-negligible role modifying *T*_N_ between samples whose thickness is significantly different. Note that a drop in *T*_N_ could be also expected from the volumetric size reduction of the sample, that is, not only the thickness but also the lateral size, due to the reduced coordination of spins on the surface^44,45^ and edges of the flakes. Our results seem to rule out the latter scenario since the volume of the flakes decreases much faster than the thickness with the centrifugation speed, and yet no major difference is observed. Finally, it was recently reported that a small strain on thin FePS_3_ flakes can significantly lower *T*_N_.^24^ A slight strain in the flakes induced while quenching *i*PrOH in the SQUID cannot be completely ruled out.

### Electron Transport Measurements

Once the magnetic properties of the thin flakes are established, hereafter we show that working electronic devices can be fabricated directly from solution with small LPE FePS_3_ flakes. Micrometer-spaced source/drain Au electrodes were fabricated by mask-less laser lithography and subsequent evaporation of Au onto a Si/SiO_2_ substrate; see Methods for fabrication details and Figure S12 in the Supporting Information for a scanning electron microscopy (SEM) image of an empty device. LPE FePS_3_ flakes can be deposited in several devices by dielectrophoresis (DEP). Dielectrophoresis consists in the directed motion of small particles under the presence of an external electrical field.^46−49^ Dielectrophoresis has been recently used to position liquid-suspended nanostructures such as nanoparticles,^48−50^ carbon nanotubes,^51,52^ and other 2D materials^26^ in between two-terminal electronic devices. In short, a microdroplet of *i*PrOH containing dispersed LPE FePS_3_ flakes is drop-casted onto a substrate containing the electrodes. Subsequently, an ac voltage (ν = 1 MHz, *V* = 10 V, *t* = 600 s) is applied between the electrodes that generates an ac electrical field. The specifically designed two-terminal tip-ended electrodes (see Figure S14 in the Supporting Information) focus the field and maximize the electrical gradient toward the central area of the gap; see Figure S14 in the Supporting Information for a numerical simulation of the electrical field gradient in the device. The flakes become polarized by the ac field and transported toward the areas with the largest squared electrical field gradient, i.e., the gaps between the electrodes;^26,31^ see Section 7 in the Supporting Information for additional details on the mathematical model describing dielectrophoresis.

**Figure 4:**
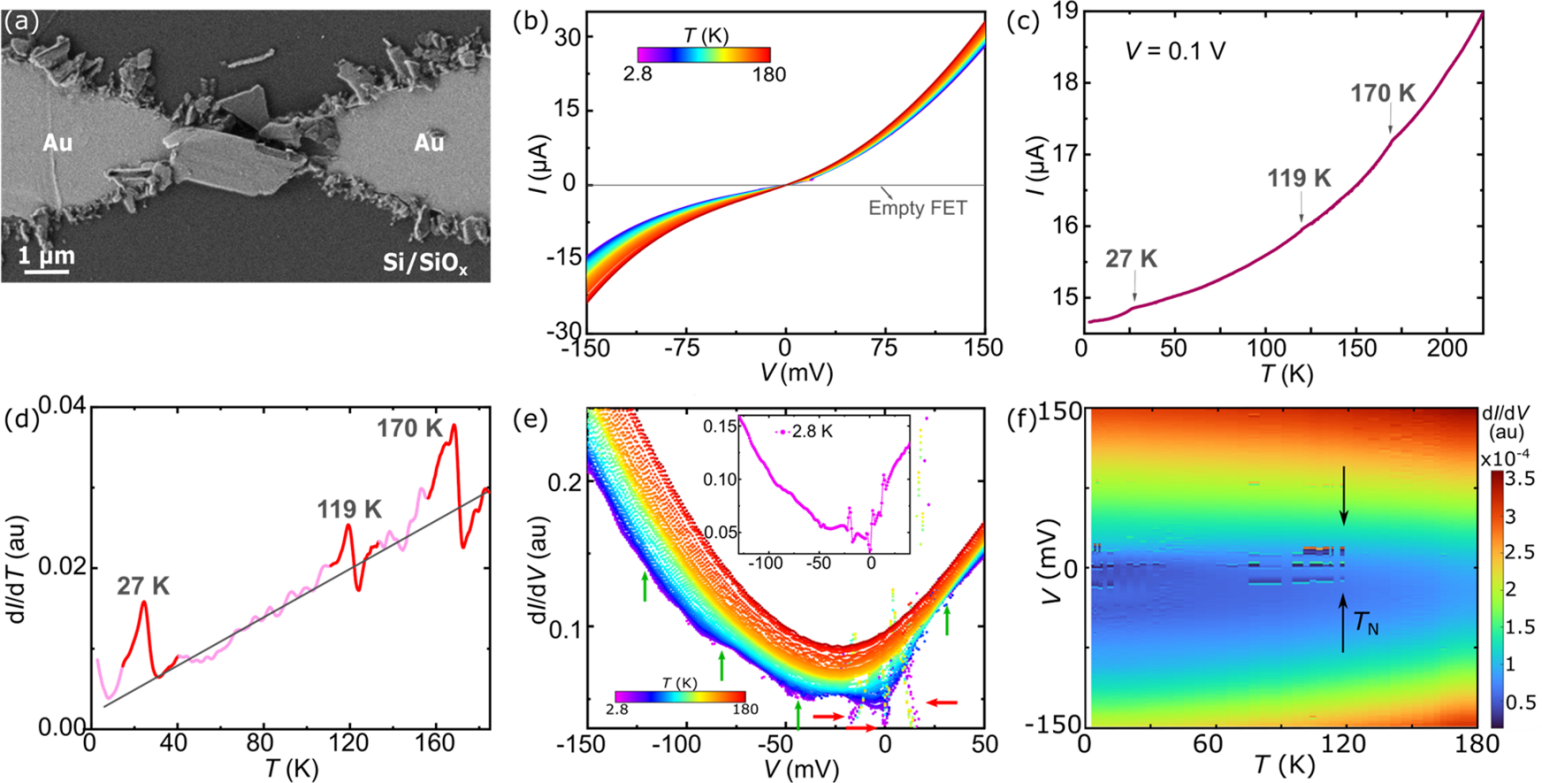
(a) SEM image of a representative electrode pair containing LPE FePS_3_ flakes (ω_1_) trapped by DEP. (b) Current–voltage (*I*–*V*) characteristics measured at different temperatures (2.8 K < *T* < 180 K). (c) Current measured on a FePS_3_ device at a fixed *V* = 0.1 V and as a function of temperature *T*. The current decreases with temperature as expected in a semiconducting material. Besides, three kinks appear at *T*_1_ = 27 K, *T*_2_ = 119 K, and *T*_3_ = 170 K. The T_1_ and T_2_ kinks roughly coincide in temperature with the magnetic transitions observed in the magnetic susceptibility (see Figure S6 in the Supporting Information). (d) First derivative (d*I*/d*T*) of the current–temperature characteristic numerically obtained from (c). The kinks in current appear as clear sinusoidal perturbations (marked in red). (e) First derivative (d*I*/d*V*) of the *I*–*V* characteristics numerically obtained from (b) at different temperatures. Three sharp, narrow perturbations (red arrows) appear at *V* = 0 V and symmetrically placed at ±18 mV below *T*_2_ and down to the lowest temperatures. Besides, a series of symmetric wider steps appear below 60 K in the ±150 mV range (green arrows). The inset shows the d*I*/d*V* curve at 2.8 K where narrow and wider excitations are clearly seen. (f) d*I*/d*V* color plot as a function of *V* and *T*. Low bias excitations are clearly observed at *V* = 0 V and symmetric ±18 mV, right below the Néel temperature (marked by the black arrows).

Figure 4a shows a SEM image of a representative device after DEP. Several FePS_3_ flakes appear filling the gap between the source and drain electrodes and decorating the electrodes’ edges. The latter, together with a slight asymmetry in the distribution of the flakes, is explained in terms of residual electrical fields between the electrodes and the underlying Si substrate.^49^ The surface far from the electrodes remains clean of flakes. The efficiency of DEP in preparing these devices contrasts with the control samples in which simple drop-casting in the absence of an electrical field has been used (see Figure S15 in the Supporting Information for a direct comparison and Figure S13 for additional SEM images). An initial electrical evidence of the formation of the FePS_3_ bridge between electrodes is the sizable current across the device after DEP, as seen in Figure 4b. The current–voltage (*I*–*V*) characteristics show an s-shape behavior characteristic of semiconducting materials and nonohmic contacts. Note that two-terminal measurements are adequate, as commonly used in other electron transport measurements in FePS_3_,^53−55^ since contact resistance is expected to vary monotonously with temperature. Variations in the total conductance between reports^53−55^ come as a result of the different dimensions of the devices and the different electrostatic environment (doping agents, etc.).

Figure 4c shows the current measured at a fixed bias *V* = 0.1 V as a function of the temperature down to 2.8 K. All temperature-dependent measurements are performed under high vacuum (∼10^–6^ mbar) in a cryo-free ICE Oxford refrigerator (see Methods). The current decreases with temperature as expected for a semiconducting material. The same trend is observed in the *I*–*V* characteristics shown in Figure 4b. Interestingly, three kinks in the conductance can be observed at temperatures *T*_1_ = 27 K, *T*_2_ = 119 K, and *T*_3_ = 170 K in Figure 4c. The kink at *T*_2_ is rather faint but clearly shows up as a sinusoidal perturbation in the first derivative of the current with temperature, shown in Figure 4d; see Figure S16 in the Supporting Information for additional examples.

Interestingly, the kink at *T*_2_ fits with the Néel temperature for the antiferromagnetic transition (Figure 3). Therefore, the magnetic transition persists in the device geometry and can be translated into a perturbation in the conductance of the material. A similar kink in the conductance has been observed for the ferromagnetic CrI_3_ and is associated with slightly different barrier heights for spin-up and spin-down electrons.^11^ On the other hand, a change in the phonon scattering at the antiferromagnetic transition could be translated into a perturbation in the current. The activation of phonon modes at the Néel temperature has been shown for FePS_3_ by Raman spectroscopy.^13,14^

The kink at *T*_1_ roughly coincides with a second magnetic transition observed at *T* = 20 K in the magnetic susceptibility (see Figure S11 in the Supporting Information). The later peak has been tentatively ascribed in the literature to a secondary magnetic ordering process in the direction perpendicular to the layers,^56^ although no clear proof is provided. Conversely, a similar, second low-temperature magnetic transition has been observed in the analogous MnPS_3_ magnetic trichalcogenide that has been associated with the unbinding of spin vortices.^57,58^

Finally, the peak at *T*_3_ does not fit with any magnetic nor structural transition reported in the bulk FePS_3_.^59^ Interestingly, a similar third peak above the Néel temperature has been observed for the analogous exfoliated MnPS_3_, but was also not observed in bulk, and has been associated with two-dimensional spin critical fluctuations.^57,60^

The low-bias electron transport spectrum is studied in more detail. Figure 4e,f shows the differential conductance (d*I*/d*V*) and the corresponding color map plot, as a function of bias voltage and at different temperatures, numerically obtained from the measurements in Figure 4b (see Figure S17 in the Supporting Information for a larger bias window). Above 119 K, the conductance grows monotonously with bias and is roughly symmetric at positive and negative biases. Interestingly, a complex spectrum of inelastic excitations appears right at the antiferromagnetic transition temperature. First, a series of sharp peaks appear at zero bias and symmetrically placed at ±18 mV (red arrows in Figure 4e); see the inset in Figure 4e for a representative d*I*/d*V* curve at the lowest temperature (2.8 K). The onset of the peaks at the Néel temperature is more clearly seen in Figure 4f. In addition, a series of step-like excitations appear below 60 K, more clearly at negative bias voltage (green arrows in Figure 4e); see Figure S18 in the Supporting Information for the second derivative of the current for clarity. These excitations could have a phononic origin, as a result of new phonon modes arising in the material.^13,14^ However, the fact that they appear right at the magnetic transition indicates that they may have a magnonic origin.^11^ Hybridization between phonons and magnons in FePS_3_ has been previously observed by Raman.^61^ Moreover, similar excitations in a similar bias window have been associated with magnon-assisted transport in the ferromagnetic CrI_3_^11^ and CrBr_3_.^62^ An in-depth analysis of the inelastic spectrum in FePS_3_ thin layers will require an alternative geometry that introduces tunnel barriers between electrodes and flake. This will be the subject of a further study.

### Covalent Functionalization of FePS_3_

The chemical modification of 2D materials with small molecules has the potential to modify their surface properties (i.e., fine-tune colloidal properties, include specific interactions, etc.) and electronic features (i.e., improve absorption, modify band gap, etc.).^63−67^ Moreover these small molecules can be used as intermediate linkers to couple 2D magnetic materials to other 2D materials with complementary properties. LPE in *i*PrOH, without the addition of exfoliating agents, provides the additional advantage of a pristine flake surface to carry out further covalent functionalization with functional molecules or even the fabrication of heterostructures.^68^

**Figure 5:**
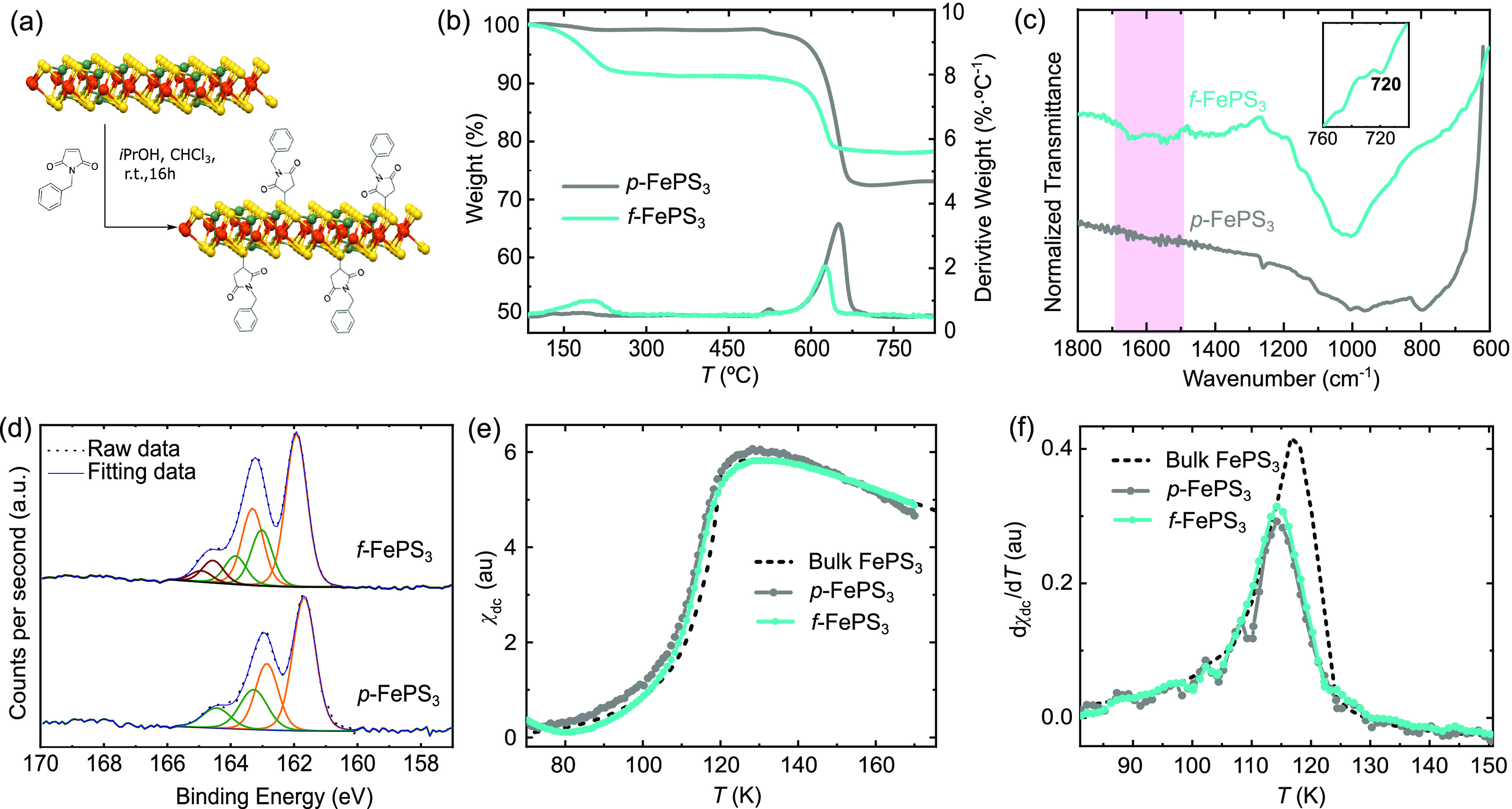
(a) Schematics of the covalent functionalization of pristine p-FePS_3_ flakes into f-FePS_3_ with *N*-benzylmaleimide. (b) Thermogravimetric analysis p-FePS_3_ and f-FePS_3_ thin flakes. A new weight loss in f-FePS_3_ is indicative of removal of organic material covalently bond to the FePS_3_. The estimated functionalization is ca. 7%. (c) ATR-FTIR spectra measured in p-FePS_3_ and f-FePS_3_. A new band at 1646 cm^–1^ in f-FePS_3_ is indicative of the covalent bond formation. (d) XPS spectra of S 2p core level of p-FePS_3_ and f-FePS_3_. Due to the formation of the C–S bond, a third component is required for the f-FePS_3_. (e) Magnetic susceptibility and (f) first derivative measured as a function of temperature in p-FePS_3_ and f-FePS_3_. The dotted line is the measurement in bulk used as a reference. No significant variation is observed in f-FePS_3_ when compared with p-FePS_3_, as expected for the low functionalization.

As a proof-of-concept, functionalization of the few-layer FePS_3_ flakes with *N*-benzylmaleimide was performed according to the thiol-maleimide “click” reaction described previously for other sulfide-based materials^68−70^ (see scheme in Figure 5a). 10 mL of 1 mM solution of *N*-benzylmaleimide in CHCl_3_ was added to 10 mL of the suspension of exfoliated FePS_3_ in *i*PrOH (sample ω_1_), and the mixture was sonicated for 5 min and stirred vigorously for 16 h at room temperature (ca. 1000 rpm). The suspension was then filtered through a polytetrafluoroethylene membrane (pore size of 0.2 μm), and the material was redispersed sonicating for 5 min in 30 mL of CHCl_3_. The colloid was filtered again and washed three times with 30 mL of CHCl_3_ to remove noncovalently linked *N*-benzylmaleimide. The material obtained through this method consists of *N*-benzylsuccinimide molecules covalently bonded to few-layer FePS_3_ by the formation of C–S bonds, and is referred in this work as f**-**FePS_3_.

Pristine (p-FePS_3_) and f-FePS_3_ were characterized through thermogravimetric analysis (TGA), X-ray photoemission spectroscopy (XPS), UV–vis electronic absorption and IR, and SQUID magnetometry. In Figure 5b, the TGA under N_2_ of p-FePS_3_ shows its main decomposition temperature at 600–700 °C, which is maintained in the functionalized sample. At lower temperatures, p-FePS_3_ shows a small (ca. 1%) weight loss between 100 and 210 °C, which can be ascribed to the elimination of adsorbed solvent. In f-FePS_3_, the low-temperature region shows a broader (100–250 °C) and more significant (ca. 8%) weight loss, which we assign to removal of the covalently bond organic material, overlapping with the solvent loss. Assuming the amount of physisorbed solvent is approximately the same for both samples, the estimated functionalization of f-FePS_3_ with *N*-benzylmaleimide is ca. 7%. Both the decomposition pattern and the degree of functionalization are comparable to what we have previously found in the covalent functionalization of franckeite.^70^Figure 5c shows the ATR-FTIR spectra of p-FePS_3_ and f-FePS_3_ in solid state at room temperature. For both materials, the trichalcogenide is characterized by the presence of an intense band at 567 cm^–1^, corresponding to the asymmetric stretching vibration of the (P_2_S_6_)^−2^ structure (ν_PS_), and a second feature at 442 cm^–1^, corresponding to the stretching vibration of the P–P single bond (ν_P–P_).^21,71^ The main IR signature of functionalization is the growth of a new low-intensity band around 1646 cm^–1^ in f-FePS_3_, assigned to the stretching vibration of the carbonyl groups of the succinimide moiety (ν_C=O_). In addition, a weak band related to the stretching vibration of the C–S single bond formed upon functionalization was detected in the ATR-FTIR spectrum of the functionalized FePS_3_ (around 720 cm^–1^, inset in Figure 5c). XPS under ultrahigh-vacuum conditions further showed the covalent anchor of the organic moiety (Figure 5d). The binding energy reference (C 1s core) was centered at 284.8 eV. The S 2p region of p-FePS_3_ fit two doublets centered at 161.7 and 163.0 eV. After the functionalization (f-FePS_3_), these bands become broader and lightly shifted (161.9 and 163.2 eV) requiring a third component, which can correspond to formation of S–C bond.^68^

Finally, the magnetism of the f-FePS_3_ flakes is explored following the procedure described above. Figure 5e compares the magnetic susceptibility of p-FePS_3_ (sample ω_1_), f-FePS_3_, and bulk powder. No significant deviation in the Néel temperature is observed when comparing p-FePS_3_ and f-FePS_3_ (see Figure 5f). This can be easily understood in terms of the low functionalization achieved for the material (ca. 7%). This functionalization yield is enough for the formation of heterostructures^68^ but has a negligible effect in the global magnetism of the flakes. This is ideal for the linking of functional molecules in the future, so that the properties of the molecular fragments can be added to the final ensemble without losing the intrinsic properties of the pristine 2D material. Further, magnetic molecules may be covalently linked to mutually modulate the magnetic properties of molecules and FePS_3_.

## Conclusions

To summarize, we show how the LPE method provides a powerful tool to study the magnetism of layered materials, including antiferromagnets. LPE exfoliation provides thousands of thin flakes suspended in a liquid media that can be further quenched and measured by SQUID magnetic measurements. As a result of this, we show a SQUID direct magnetic evidence of the antiferromagnetic transition in FePS_3_ few-layer flakes in solution. Moreover, LPE provides some control over the average flake thickness. A clear reduction of the Néel temperature with the flake thickness is observed in the magnetic SQUID measurements.

LPE FePS_3_ flake-based electronic devices can be prepared by dielectrophoresis. Low-temperature, electron-transport measurements show a sizable conductance in the device concomitant with a conductance anomaly and the appearance of potential magnon-phonon hybrid states right at the Néel temperature (antiferromagnetic order transition) of the FePS_3_. In addition, a secondary magnetic transition, observed in SQUD measurements, appears in the current at the lowest temperatures. The combination of these results, therefore, shows that thin and small LPE FePS_3_ few-layer flakes preserve their magnetic properties and can be implemented in electronic devices.

Finally, we show the covalent functionalization of the FePS_3_ few-layer flakes in solution obtained by LPE. As a proof-of-concept, small *N*-benzylmaleimide molecules are covalently bound to LPE FePS_3_ thin flakes. The magnetic properties of the flakes are preserved; therefore, this technique can be an interesting path for the mass functionalization of 2D magnetic materials.

## Methods

### Chemicals and Reagents

Bulk FePS_3_ material is obtained from Ossila (product number M2207C1). The same material is used for all experiments. The solvent (*i*PrOH) used for LPE process is purchased from Scharlab Chemicals S.L. and used without further purification.

### Preparation of FePS_3_ Liquid Suspension

Starting from bulk, FePS_3_ crystals are ground in an agate mortar until a fine black powder is obtained. FePS_3_ powder (10 mg) weighed in a precise scale is dispersed in *i*PrOH (10 mL) in a glass vial. In this way, the initial dispersion at 1 mg/mL concentration is obtained. Thereafter, the liquid phase exfoliation is carried out. The dispersion is exposed to ultrasound irradiation for 40 min in an ultrasonic bath (Fisher Scientific FB 15051; 37 kHz, 280 W, ultrasonic peak max. 320 W, standard sine-wave modulation) connected to a cooling system maintaining the water bath temperature at 20 °C. The resulting black suspension is centrifuged according to size-selection cascade centrifugation process (Beckman Coulter Allegra X-15R, FX6100 rotor, radius 9.8 cm) which involves several centrifugation steps (30 min per step) with gradually increasing centrifugal acceleration (ω_1–4_ = 1000 (110), 3000 (986), 5000 (2739), and 7000 (5369)) rpm (g)). In each step, a black sediment and a gray supernatant are obtained. The supernatant is carefully isolated from the solid, where part of it is kept as a final sample, while the rest is centrifuged under a higher rotational speed. At the end of the cascade process, four sediment fractions and four supernatant fractions (LPE FePS_3_ 1000, 3000, 5000, and 7000 rpm) are obtained. The suspensions remained colloidally stable for 72 h, after which they progressively deposited. Nevertheless, the flakes could easily be redispersed by 1–2 min bath sonication.

To determine the concentration of the exfoliated material after each centrifugation step (ω_1–4_), the collected supernatants were filtered in PTFE 0.2 μm membranes. Thereafter, the mass of the exfoliated material is weighted, and the volume in which it was dispersed is measured. The concentration values for each sample are provided in Table S1.

### Raman Spectroscopy

Raman spectra of LPE FePS_3_ samples are taken on solid-state samples prepared after filtering the liquid dispersion to obtain a film of material that is deposited on a glass slide. Raman study is recorded with a Bruker Senterra confocal Raman microscope (Bruker Optic, Ettlingen, Germany, resolution 3–5 cm^–1^) using the following parameters: objective NA 0.75 × 50; laser excitation: 785 nm, 1 mW, 2 co-additions, 1 s integration time. The spectra result from the average of 144 measurements acquired on a 14 μm × 14 μm area covered with flakes; see optical image of the map area in the Supporting Information. For temperature-dependent Raman spectroscopy measurements, a Linkam electrical probe station (HFS600E-PB4 stage, Linkam Scientific Instruments) equipped with a LNP 95 liquid nitrogen cooling system was coupled to the Raman equipment stage.

### Atomic Force Microscopy

Dried AFM samples are prepared by spin-coating (Laurell Technologies, WS-400BZ-6NPP/LITE Spin Coater) of the corresponding dispersion on mica foil (samples ω_1–4_ = 1000, 3000, 5000, and 7000 rpm) and dried in air. AFM images are acquired using commercial AFM systems (NT-MDT Ntegra Prima and JPK Nanowizard 2) in semicontact (dynamic) mode in ambient conditions. NT-MDT NSG01 silicon cantilevers, with typical values of 5.1 N·m^–1^ spring constant and 150 kHz resonant frequency, are employed in all the cases. The resulting images processing and the statistical analysis were done using WSxM software^72^ (version 5.0, Nanotec Electronica S.L., Spain).

### Dielectrophoretic Process

The DEP technique was performed in a Lakeshore Cryogenics (Model PS-100 Tabletop) probe station, equipped with a FeelTech FY3200S Dual-channel Arbitrary Function Signal Generator, applying the following parameters: *V*_ac_ = 10 V, ν = 1 MHz, and *t* = 10–30 min; see Section 7 in the Supporting Information for a detailed description of the technique (solvent, geometry and mathematical model).

### Scanning Electron Microscopy

SEM images are recorded by the inlens detector of a ZEISS SIGMA VP field emission scanning electron microscope (FE-SEM) from Carl Zeiss Microscopy. The acceleration voltage is 2 kV, and the current applied is around 45 pA.

### Transmission Electron Microscopy

TEM images were obtained in a JEOL JEM 2100 microscope operating at 200 kV. Images were recorded on a CCD ORIUS SC1000 (Model 832) camera. A few drops of LPE FePS_3_ dispersion were deposited onto a 200 square mesh grid covered by holey carbon. After solvent evaporation in air, the grid was ready to use.

### X-ray Photoelectron Spectroscopy

XPS measurements were performed with an Axis Supra spectrometer (Kratos Analytical Ltd., UK). The samples were drop casted onto an Au substrate; three spectra were acquired with a charge neutralization beam to avoid differential charging of samples. The quantitative composition was determined from detailed spectra taken at the pass energy of 80 eV. A lower pass energy of 20 eV was used to attain well-resolved spectra for fitting. The deconvolution of XPS spectra to individual components was done in the Casa XPS 2.3.17 software. For the fitting, the Shirley-type background subtraction was used, and all curves were defined as 30% Lorentzian, 70% Gaussian. Besides, constrains of the full width at a half-maximum (fwhm) and the peak positions were applied. Binding energy calibration was based on adventitious carbon at 284.8 eV.

### Electron Transport Measurements

The current–voltage and current–temperature characteristics were obtained in vacuum conditions in a Cryogen-Free Closed Cycle 4K Cryostat (Dry Ice 4K System, ICE Oxford) equipped with a Lakeshore Temperature Cryogenic Controller Model 336, a Keithley 2450 digital source-meter unit, and a Tenma 72-270 Programmable DC power supply (60 V, 3 A).

### Magnetic Measurements

Magnetic susceptibility measurements were performed in a Quantum Design MPMS-5S SQUID magnetometer mounted in a low-temperature cryostat, under a 1000 Oe field. Each sample was secured inside a diamagnetic capsule. Samples with low concentration were left to evaporate for some time to increase concentration and magnetic signal.

### Device Fabrication Details

The multielectrode devices are fabricated via laser mask-less optical lithography and thermal evaporation of Cr/Au (5/80 nm) electrodes on a highly doped silicon substrate capped with a 300 nm-thick insulating SiO_2_ layer, used as common back-gate electrode. Initially, Si/SiO_2_ wafers are cleaned using *i*PrOH and acetone to remove any traces of organic, ionic, and metallic impurities. Then, AZ1505 positive photoresist is spin coated at 5000 rpm for 1 min onto the surface followed by baking at 90 °C for 1 min to form a 450 nm resist layer. The electrodes and pads are defined by exposing the surface to UV light using a Heidelberg Instruments DWL66 fs laser writer of 405 nm (h-line) with 300 mJ/cm^2^ dose. The pattern is subsequently developed with AZ-351B. Thereafter 5 nm Cr and 80 nm Au layers are deposited using Ecovac e-beam evaporation by Angstrom Engineering. A lift-off process in acetone/*i*PrOH/deionized water removes the excess metallic material. The finger-shaped electrodes are connected to common Au pads that allow performing simultaneous dielectrophoresis to all the devices. The size of the gap created between a pair of electrodes ranges between 750 nm and 1 μm from device to device. The devices are annealed at 300 °C for 8 h after the fabrication.

## Supporting Information Available

Raman spectroscopy, TEM images, AFM images and statistical details, additional magnetic measurements, details about dielectrophoresis, additional SEM images, additional electron transport measurements (PDF)
