# Supplementary material for: Direct Magnetic Evidence, Functionalization, and Low-Temperature Magneto-Electron Transport in Liquid-Phase Exfoliated FePS3
Source: ACS Nano. 2023 Jan 18;17(3):3007–18. doi: 10.1021/acsnano.2c11654 (PMC9933618; doi:10.1021/acsnano.2c11654)
Supplement: Supplementary file 1 — nn2c11654_si_001.pdf [file nn2c11654_si_001.pdf]

SUPPORTING INFORMATION. Direct magnetic evidence, functionalization and low-temperature magneto-electron transport in liquid-phase exfoliated $FePS_3$

Lucía Martín-Pérez[1], Samara Medina Rivero[1], Manuel Vázquez Sulleiro[1], Alicia Naranjo[1], I. Jénnifer Gómez[2], María Luisa Ruíz-González[3], Andres Castellanos-Gomez[4], Mar Garcia-Hernandez[4], Emilio M. Pérez[1*] and Enrique Burzurí[5,1*]*

[1]IMDEA Nanociencia C/Faraday 9 Ciudad Universitaria de Cantoblanco, 28049 Madrid, Spain.

[2] Department of Condensed Matter Physics, Faculty of Science, Masaryk University, Kotlářská 2, 61137 Brno, Czech Republic

[3]Departamento de Química Inorgánica, Universidad Complutense de Madrid, 28040 Madrid, Spain

[4]2D Foundry, Instituto de Ciencia de Materiales de Madrid (ICMM), Consejo Superior de Investigaciones Científicas (CSIC), 28049 Madrid, Spain

[5]Departamento de Física de la Materia Condensada and Condensed Matter Physics Center (IFIMAC), Universidad Autónoma de Madrid, 28049 Madrid, Spain

E-mail: enrique.burzuri@uam.es, emilio.perez@imdea.org marmar@icmm.csic.es

Index

1. Determination of the concentration ... 3

2. Additional Raman spectroscopy details ... 3

3. Additional Transmission Electron Microscopy (TEM) images of LPE $FePS_3$ few-layer flakes. ... 8

4. Additional Atomic Force Microscopy (AFM) images and statistical details ... 8

5. Additional magnetic measurements. ... 13

6. Additional Scanning Electron Microscopy (SEM) images of LPE $FePS_3$-based devices after DEP assembly. ... 15

7. Additional details of the dielectrophoresis technique ... 17

8. Electron transport measurements ... 21

9. References ... 22

1. Determination of the concentration

The concentration of exfoliated material in each sample is calculated from the supernatant collected after the centrifugation process. As indicated in the manuscript, we started from a dispersion of ground $FePS_3$ (10 mg) in *i*PrOH (10 mL) ($C_i = 1$ mg·mL^{-1}). This initial dispersion is sonicated and centrifuged following a cascade process, where a portion of the supernatant is extracted in each stage, giving rise to the different samples (ω_{1-4}).

The collected supernatants were filtered (in PTFE 0.2 um membranes) to determine the mass of exfoliated material and the volume in which it was dispersed, allowing us to know the real concentration of the exfoliated material for each sample, Table S1.

Table S 1 Concentration of exfoliated material for each sample (ω_{1-4}) calculated from the supernatants collected after LPE process (see main text).

Sample	C_f [mg·ml^{-1}]
ω_1 (1000 rpms)	0.2
ω_2 (3000 rpms)	0.17
ω_3 (5000 rpms)	0.09
ω_4 (7000 rpms)	0.07

2. Additional Raman spectroscopy details

Figure S1 shows the normalized Raman spectra of both (a) bulk and (b) ω_1 (1000 rpms) liquid-phase exfoliated $FePS_3$ samples. The figure shows the Lorentzian fit of each peak (colored-filled areas) and the resulting cumulative fit (dotted line). The band shift values obtained in said Lorentzian setting are shown in table S2. The final spectra result from the average over 144 spectra measured in a 14x14 μm area covered by $FePS_3$. See Figure S2 for an optical image of the mapping area and the distribution of points.

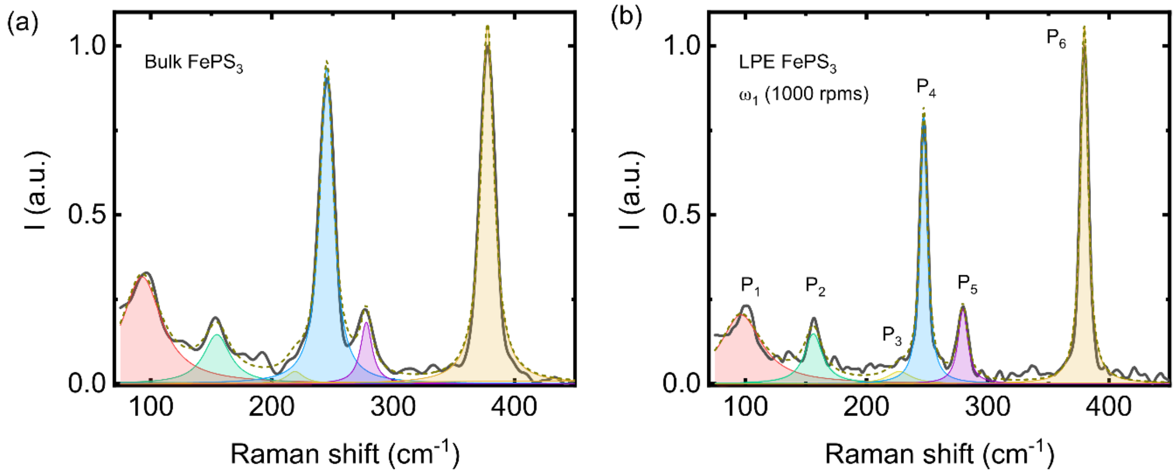

Figure S 1 Normalized Raman spectra (λ_{exc} = 785 nm) of (a) bulk and (b) ω_1 (1000 rpms) LPE $FePS_3$ in solid state at room temperature. Colored-filled areas and dotted lines correspond to the peaks deconvolution fit and cumuliative fit spectrum, respectively, using the Loreztian function. The position obtained on the peaks is shown in table S2.

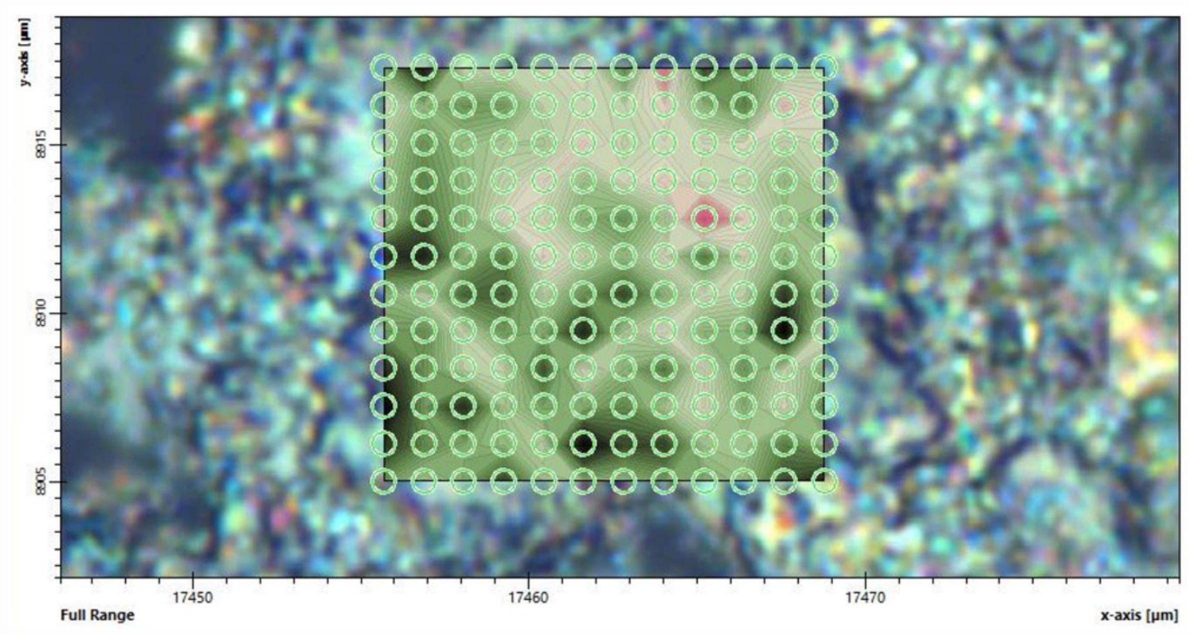

Figure S 2. Optical image of LPE $FePS_3$ (ω_1) in solid-state prepared after filtering the liquid dispersion to obtain a film of material that is deposited on a glass slide. The final spectrum is the average over 144 Raman measurements in the positions marked by the dots in a 14µmx14µm area.

Table S2. Raman shifts of bulk and ω_1 (1000 rpms) LPE $FePS_3$ samples assigned by deconvolution using Lorentzian function (R^2>0.98).

$FePS_3$	$P_1(cm^{-1})$	$P_2(cm^{-1})$	$P_3(cm^{-1})$	$P_4(cm^{-1})$	$P_5(cm^{-1})$	$P_6(cm^{-1})$
Bulk	92.8 ± 0.5	154.6 ± 0.8	219.0 ± 1.5	245.2 ± 0.08	277.8 ± 0.4	377.8 ± 0.07
LPE ω_1 (1000 rpms)	96.4 ± 0.6	157.2 ± 0.4	228.5 ± 1.5	247.2 ± 0.06	279.4 ± 0.2	379.6± 0.04

Figures S3 and S4 show the normalized Raman spectra of ω_2 (3000 rpms) and ω_3 (5000 rpms) liquid-phase exfoliated $FePS_3$ samples respectively.

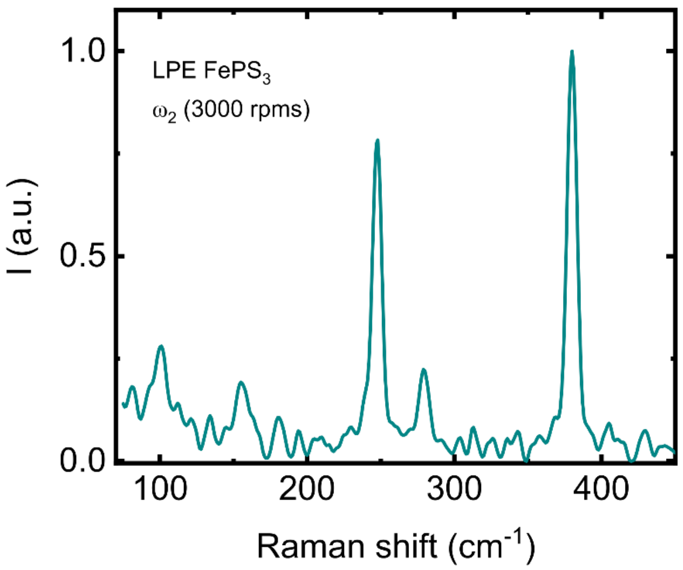

Figure S 3 Normalized Raman spectra (λ_{exc} = 785 nm) of ω_2 (3000 rpms) LPE $FePS_3$ in solid state at room temperature.

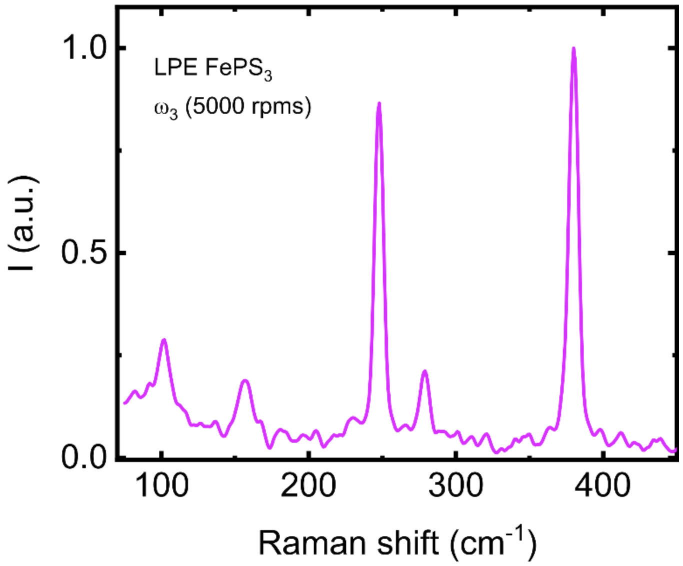

Figure S 4 Normalized Raman spectra (λ_{exc} = 785 nm) of ω_3 (5000 rpms) LPE $FePS_3$ in solid state at room temperature.

Figure S5 shows the position of peaks P_4 and P_6, two bands attributed to the molecular-like vibrations from $(P_2S_6)^{4-}$ bipyramid structures, as a function of the centrifugation speed. Larger centrifugation speeds lead to a slight P_4 redshift and P_6 blueshift associated with the thinning down of the sample as shown by Lee *et al.*[1]

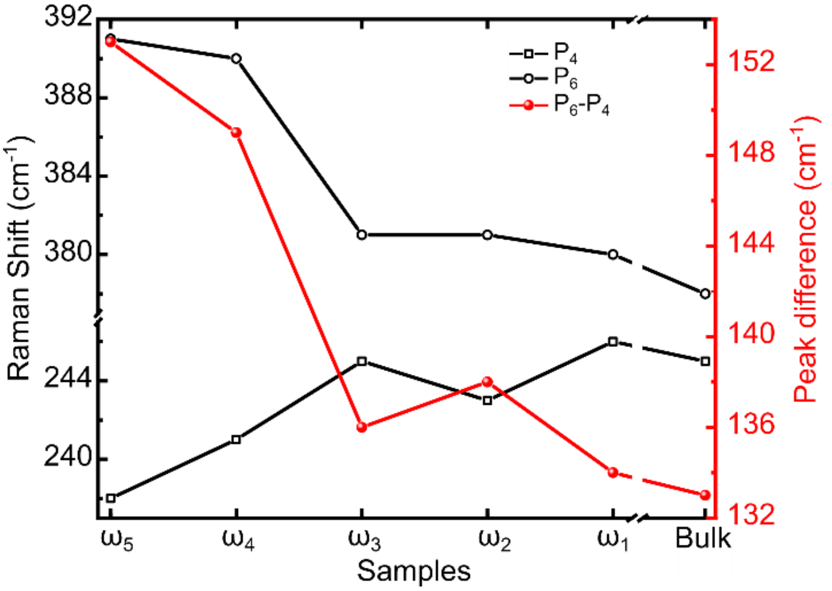

Figure S 5. P_4 and P_6 peak position and their difference (P_6−P_4) as a function of the centrifugation speed (ω).

3. Additional Transmission Electron Microscopy (TEM) images of LPE $FePS_3$ few-layer flakes.

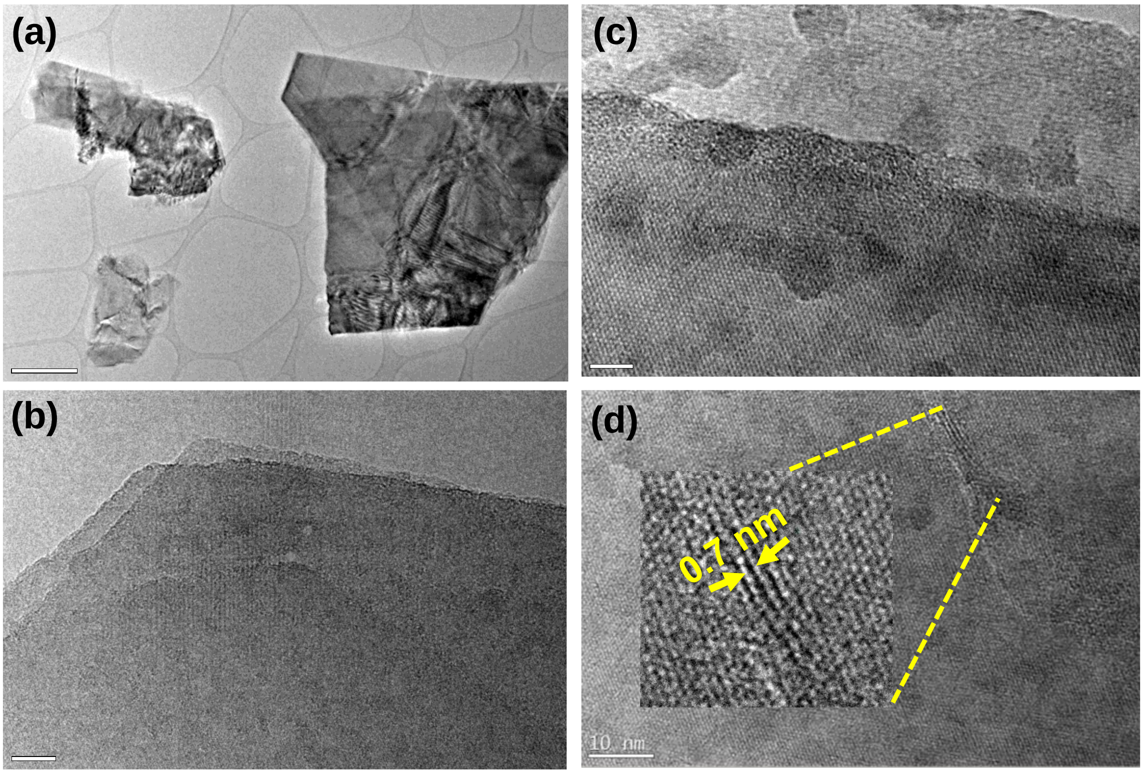

Figure S 6 a) Low magnification TEM images of several agglomerate of $FePS_3$ flakes; (b) Enhanced detail in which two layers are distinguishable at the edge; (d) Overlapping of several layers: (d) Interior part where the enhanced detail shows the blending of the layers; the interlayer distance of 0.7 nm in agreement with the c parameter of the monoclinic $FePS_3$ cell.

4. Additional Atomic Force Microscopy (AFM) images and statistical details

Figures S7, S8, and S9 (right column) shows the $FePS_3$ flakes automatically identified and selected by the WSxM software[2] for samples (a) ω_1 – 1000 rpm, (b) ω_2 – 3000 rpm, (c) ω_3 – 5000 rpm and (d) ω_4 – 7000 rpm. A minimum flake area of 0.002 μm^2 is set as boundary. The thickness, perimeter and area distribution of the selected flakes are shown in the left column of Figures S7, S8 and S9, respectively.

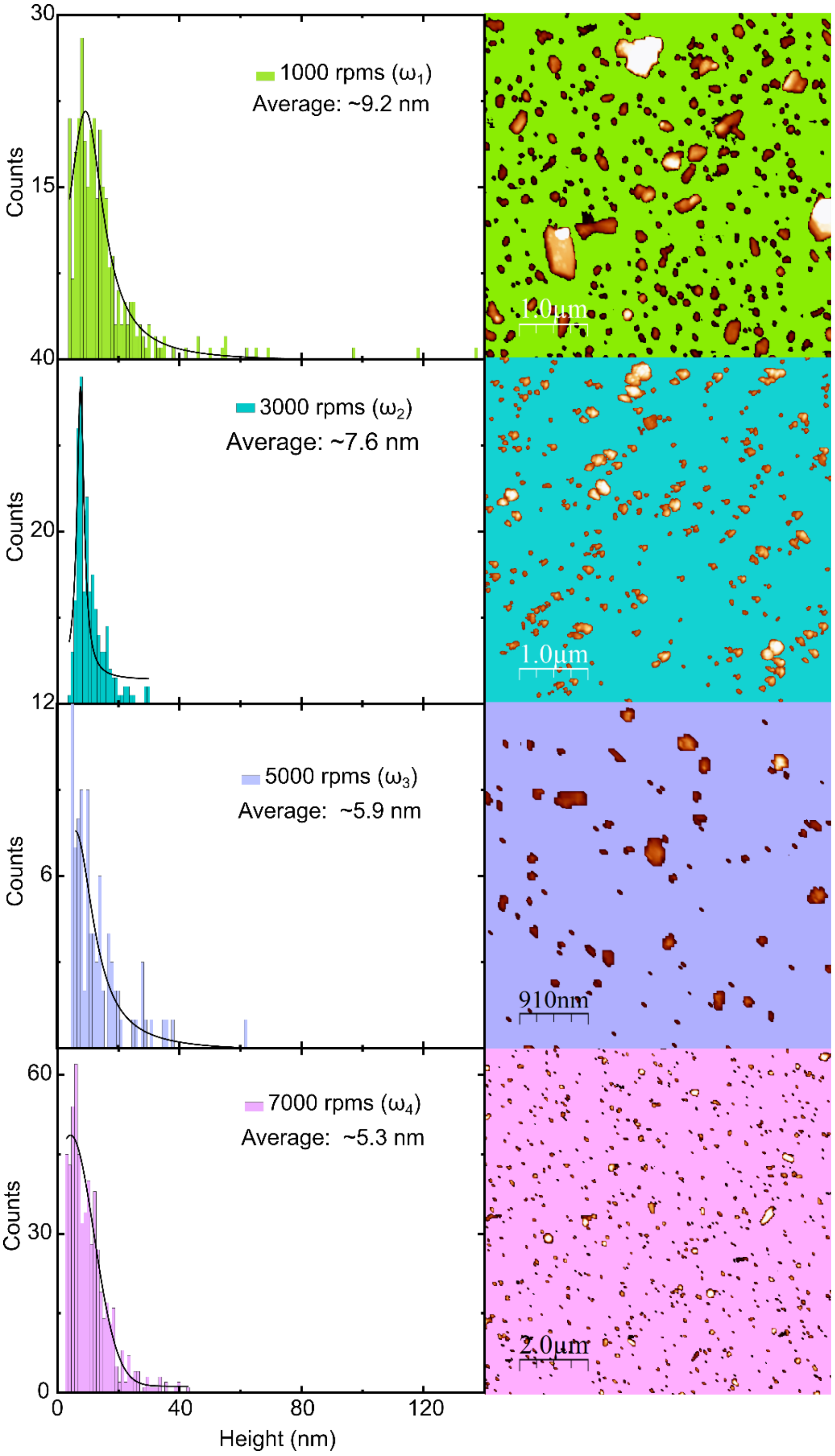

Figure S 7 AFM study of LPE $FePS_3$ few-layer flakes prepared by spin-coating the corresponding dispersion on mica foil and dried in air. Left: Flake thickness (height) distribution obtained in samples (a) ω_1, (b) ω_2, (c) ω_3 and (d) ω_4. Right: Flake selection, performed considering a minimum flake area of 0.002 μm^2.

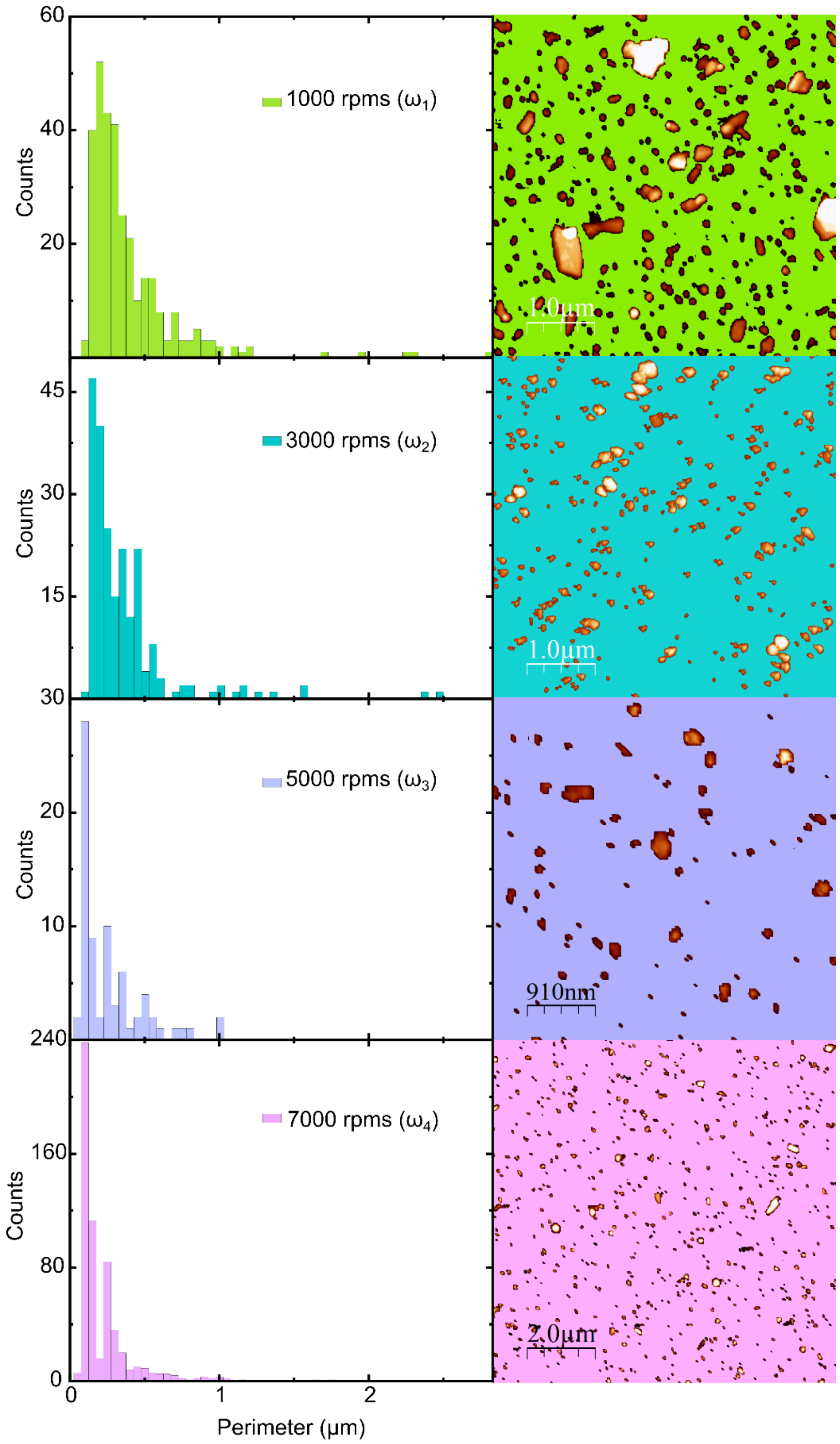

Figure S 8 AFM study of LPE $FePS_3$ few-layer flakes prepared by spin-coating the corresponding dispersion on mica foil and dried in air. Left: Flake perimeter distribution obtained in samples (a) ω_1, (b) ω_2, (c) ω_3 and (d) ω_4. Right: Flake selection, performed considering a minimum flake area of 0.002 μm^2.

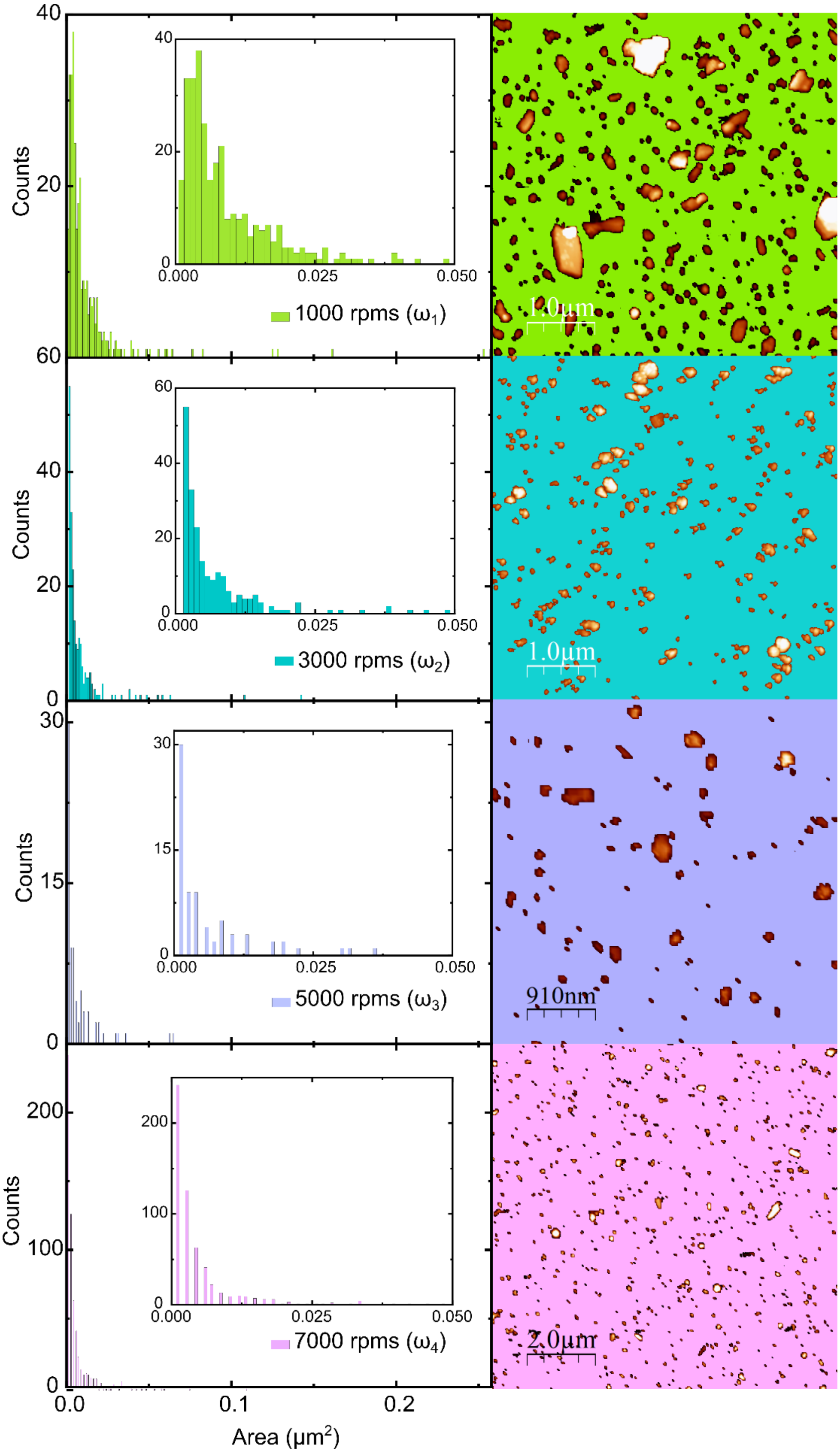

Figure S 9. AFM study of LPE $FePS_3$ few-layer flakes prepared by spin-coating the corresponding dispersion on mica foil and dried in air. Left: Flake area distribution obtained

in samples (a) ω_1, (b) ω_2, (c) ω_3 and (d) ω_4. The insets show a zoom on the lower area distribution zone. Right: Flake selection, performed considering a minimum flake area of 0.002 μm^2.

5. Additional magnetic measurements.

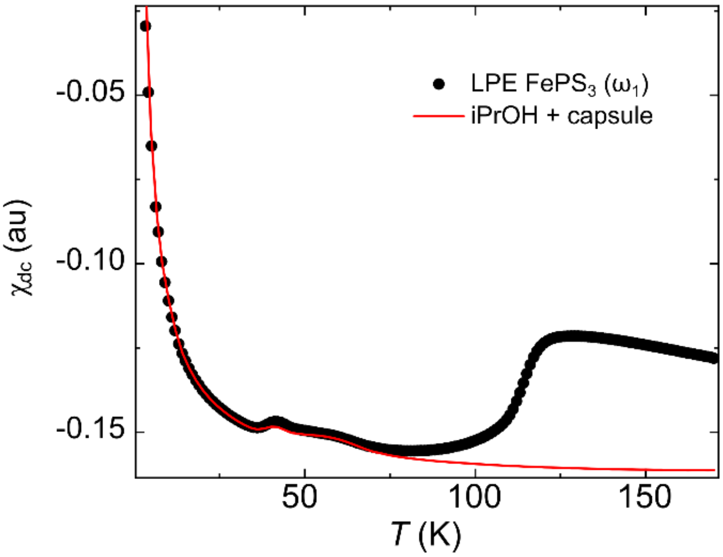

Figure S 10. Raw magnetic susceptibility measured as a function of temperature in an exfoliated ω_1 sample (black dots). The red line is the magnetic susceptibility measured in a reference plastic capsule containing isopropanol. The susceptibility in Figure 3 of the main text is the result of the subtraction.

Figure S10 shows the raw magnetic susceptibility measured in a capsule containing the LPE $FePS_3$ suspended in *i*PrOH (black dots). The red line corresponds to the magnetic susceptibility measured in a test sample where the capsule contains only iPrOH (without $FePS_3$ flakes). The contribution of iPrOH and capsule to the magnetic susceptibility is a paramagnetic component

at low temperatures and a small anomaly at around 50K. The susceptibility shown in Figure 3 in the main text results from the subtraction of this component.

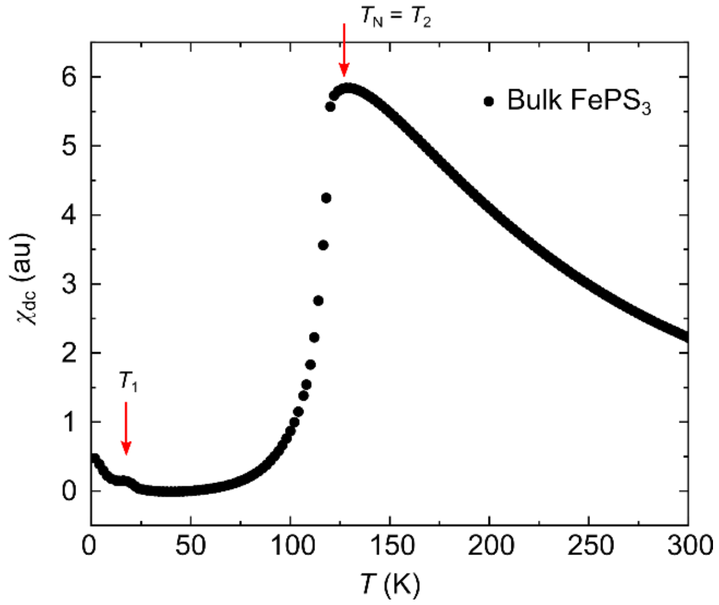

Figure S 11. Magnetic susceptibility measured as a function of temperature in a bulk reference sample. A small magnetic transition at T_1 = 20 K is observed at low temperatures in addition to the antiferromagnetic transition at T_N = T_2 = 118 K.

6. Additional Scanning Electron Microscopy (SEM) images of LPE $FePS_3$-based devices after DEP assembly.

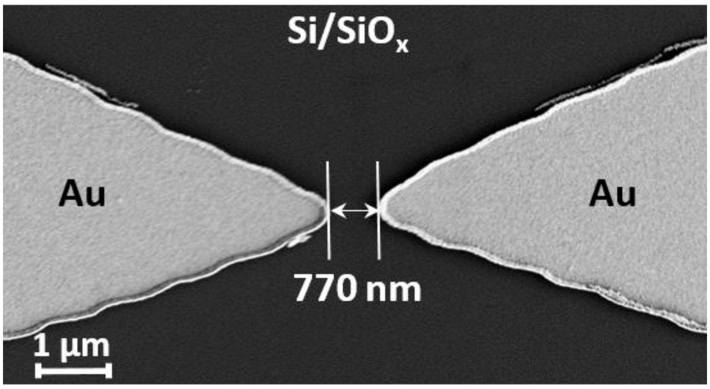

Figure S 12. Scanning Electron Microscopy (SEM) image of a representative empty source-drain electrode pair before DEP. The distance between the electrodes in the devices ranges between 750 nm and 1 µm.

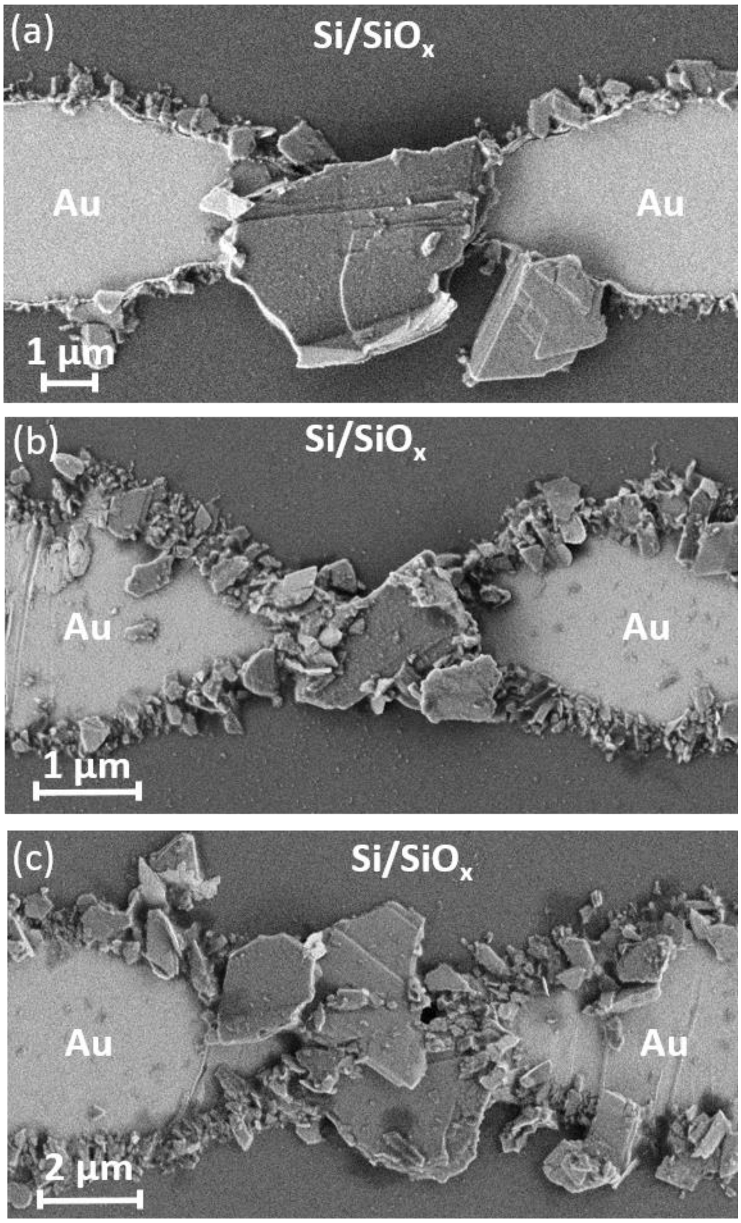

Figure S 13.Scanning Electron Microscopy (SEM) images of three representative source-drain electrode pair containing LPE $FePS_3$ flakes (ω_1) trapped by DEP. Several flakes appear filling the gap between the electrodes, forming a continuous path between them.

7. Additional details of the dielectrophoresis technique

The liquid-phase exfoliated $FePS_3$ flakes can be positioned between metallic electrodes, directly from solution, by dielectrophoresis (DEP). Dielectrophoresis consists in the directed motion of nano-objects in the presence of an ac electrical field. The electrical field polarizes the nano-objects, and exerts a dielectrophoretic force F_{DEP} that, for oblate ellipsoidal particles with large aspect ratios, can be expressed as:[3]

$$\langle F_{DEP} \rangle \propto V_p \varepsilon_m Re\left[\frac{\varepsilon_p^* - \varepsilon_m^*}{\varepsilon_m^*}\right] \nabla |E|^2 \qquad \text{Eq. 1}$$

where V_p is the volume of the particles; ε_p^* and ε_m^* are respectively the complex permittivities of the particles and the suspension medium; and $\boldsymbol{E}$ is the non-uniform electric field.

The DEP force is therefore proportional to:

(1) The nanoflake volume.

(2) The real part of the Clausius-Mossoti factor: $\left[\left(\varepsilon_p^* - \varepsilon_m^*\right)/\varepsilon_m^*\right]$ that is related with the polarizability of the particles and solvent. Besides, the sign of this factor will determine which component becomes polarized. *i*PrOH has been used before as solvent with other 2D materials due to its relatively weak polarizability.[3]

(3) The electrical field distribution and the gradient of the electrical field.

A careful design of the electrodes is therefore required to focus the electric field within the gap between the electrodes and to avoid perturbations to the gradient of the electrical field towards the gap by other parts of the circuit. Figure S14a shows the squared electrical field (E^2) distribution for two tip-ended electrodes calculated by finite elements analysis software. See

Ref.[3] for details on the simulation. Besides Figure S14b and S14c show the E^2 and $\nabla|E|^2$ profiles taken along the axis perpendicular to the electrodes axis and that crosses the gap area (red dashed line in Figure S14a). The electrical field is maximum within the gap between the electrodes. The gradient is directed towards the gap where it becomes zero. The flakes are therefore trapped once they reach the inter-electrode space. This allows the controlled accumulation of material by graduating the time of dielectrophoresis and concentration of flakes in the liquid phase exfoliation.

Figure S15 shows three different examples of this controlled accumulation of material. Figure S15a shows an SEM image of the electrodes after drop-casting an *i*PrOH droplet containing LPE $FePS_3$ flakes and in the absence of an electrical field, that is, without dielectrophoresis. The flakes are randomly distributed over the surface without a preference for the gap between the electrodes. In contrast, Figure S15b shows that by activating the ac electric field, the flakes tend to accumulate in the area predicted in Figure S14. By further increasing the flake concentration in the solvent and/or the intensity of the electrical field (time and voltage) a further accumulation of material between the electrodes can be obtained, as seen in Figure S15c.

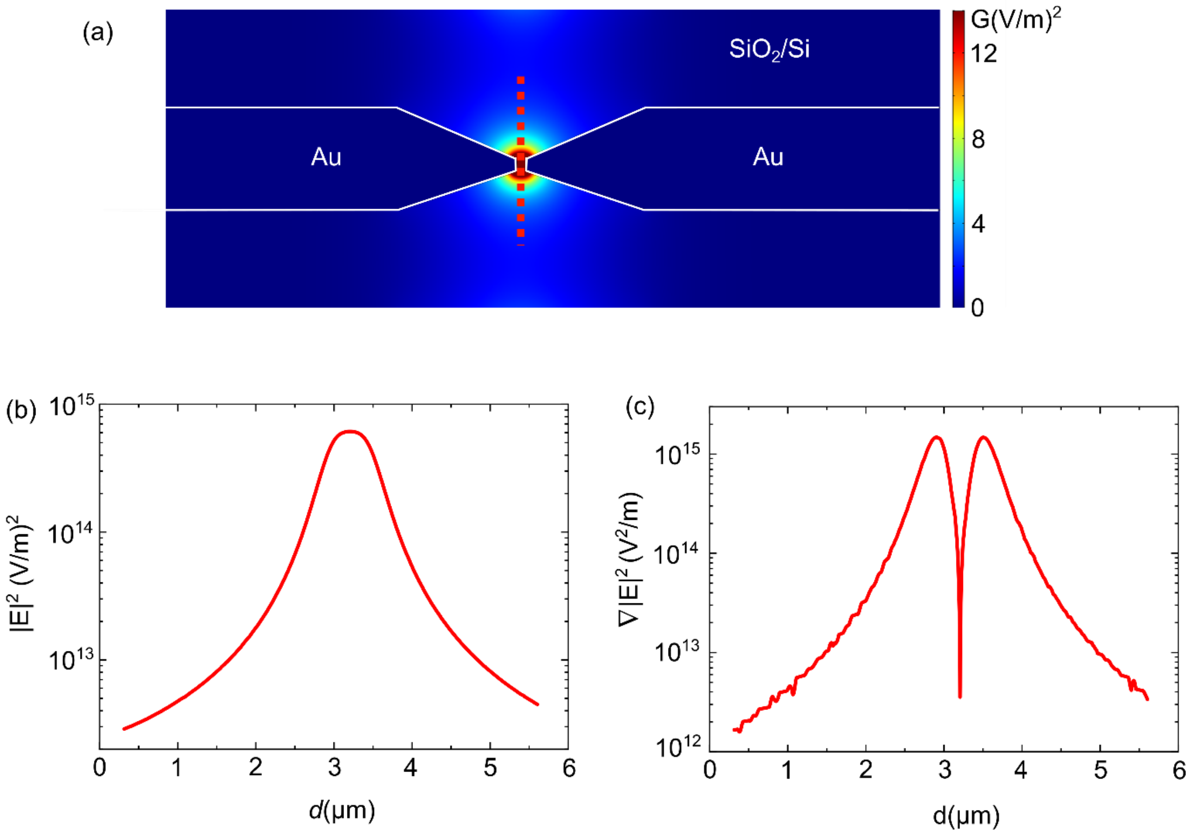

Figure S 14. (a) Squared electrical field distribution (E^2) color map simulated via finite element analysis at V =10 V. The dimensions simulate the real devices described in the main manuscript. The electrical field is maximized in the gap between electrodes. The gradient of the electrical field ($\nabla|E|^2$) points to and increases toward that area. The electrodes' edges are highlighted in white for clarity. (b) Squared electrical field (E^2) and (c) $\boldsymbol{\nabla}|E|^2$ along the axis perpendicular to the electrodes and across the gap (red dashed line in a).

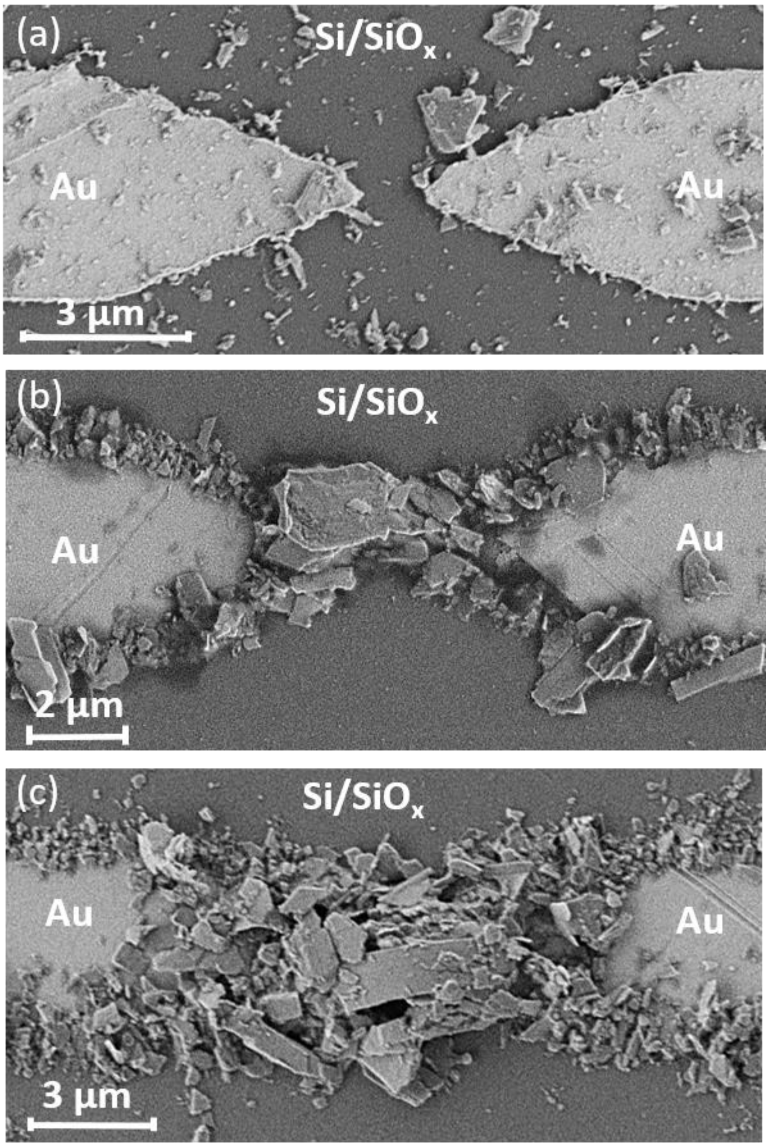

Figure S 15. *Scanning Electron Microscopy (SEM) images of three representative source-drain electrode pairs after drop-casting iPrOH containing LPE* $FePS_3$ *flakes: (a) In the absence of an applied voltage (no DEP), (b) by applying V = 10 V, t =10 min, f = 1 MHz (the conditions in the main manuscript) and (c) by doubling the time to 20 min (V = 10 V, f = 1MHz).*

8. Electron transport measurements

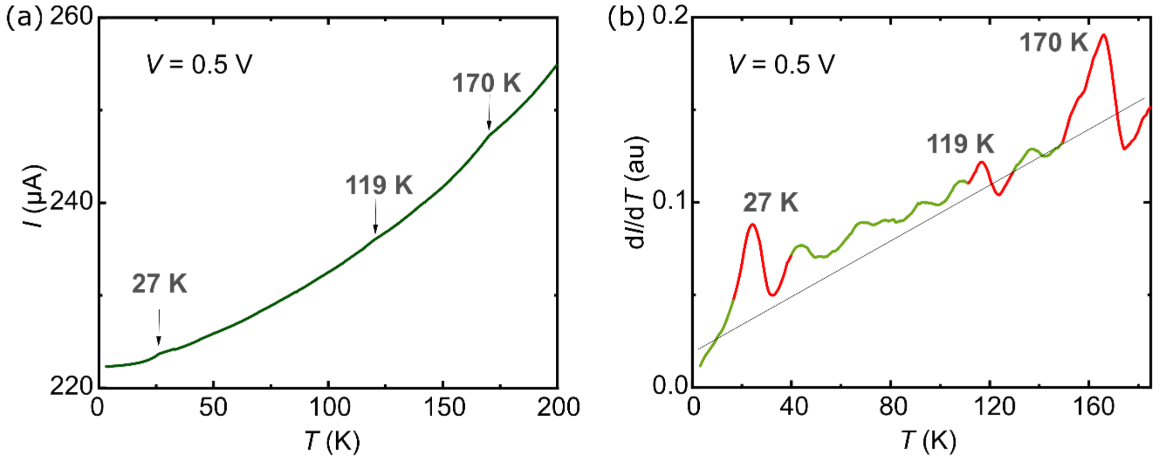

Figure S 16 Temperature-dependent current measured on an additional $FePS_3$ (ω_1) device at V = 0.5 V. Three kinks in current appear at T_1 = 27 K, T_2 = 119 K and T_3 = 170 K. (b) First derivative (dI/dT) of the current-temperature characteristic numerically obtained from (a). The kinks are seen as sinusoidal perturbations, marked in red.

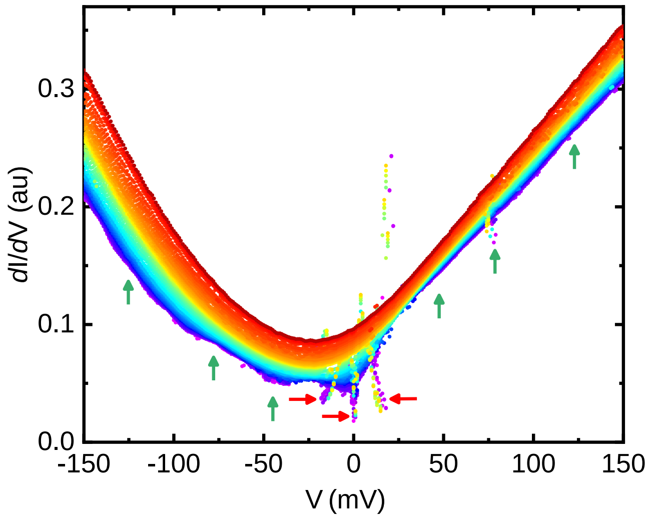

Figure S 17 First derivative (dI/dV) of the current-voltage characteristics measured at different temperatures (2.8 K < T < 180 K). Three sharp, narrow perturbations (red arrows) appear at V = 0 V and symmetrically placed at ±18 mV below T_2 (119 K) and down to the lowest

temperatures. Besides a series of wider steps appear below 60 K in the ±150 mV range (green arrows).

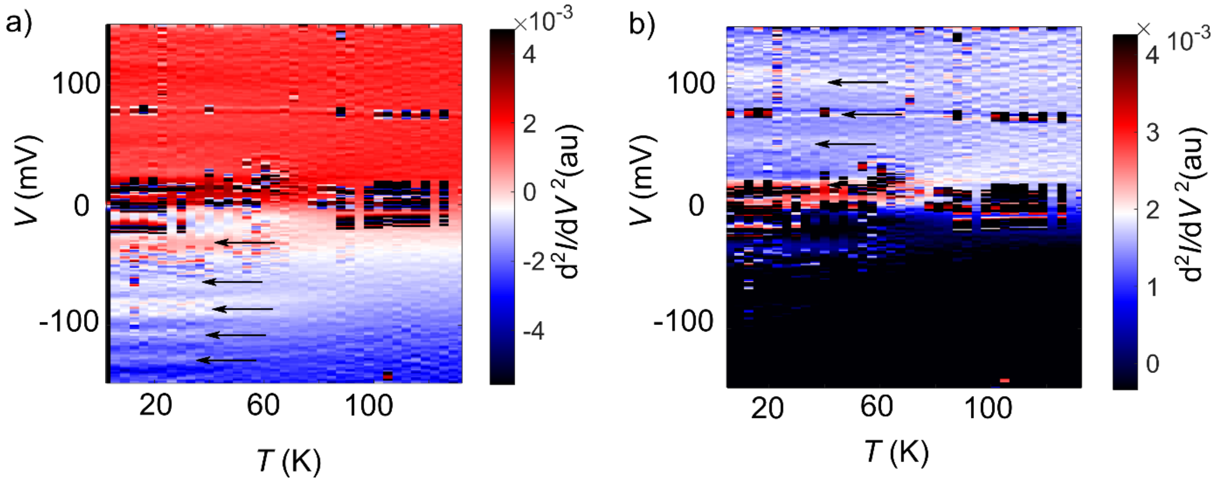

Figure S 18(a,b) Color plot of the second derivative (d^2I/dV^2) as a function of V and T (2.8 K < T < 180 K), obtained by numerical differentiation of the dI/dV data in Figure 4f of the main text. The wide steps (green arrows in Figure S17) appear as clear peaks below 60 K (marked by the black arrows). The color contrast is adjusted to optimize the visualization of the peaks at a) positive and b) negative bias voltage respectively.

9. References

1. Lee, J.-U. *et al.* Ising-Type Magnetic Ordering in Atomically Thin FePS 3. *Nano Lett.* **16**, 7433–7438 (2016).

2. Horcas, I. *et al.* WSXM: A software for scanning probe microscopy and a tool for nanotechnology. *Rev. Sci. Instrum.* **78**, 013705 (2007).

3. Burzurí, E. *et al.* Simultaneous assembly of van der Waals heterostructures into multiple nanodevices. *Nanoscale* **10**, 7966–7970 (2018).
